# Reflecting on Existential Threats Elicits Self-Reported Negative Affect but No Physiological Arousal

**DOI:** 10.3389/fpsyg.2020.00962

**Published:** 2020-05-29

**Authors:** Eefje S. Poppelaars, Johannes Klackl, Daan T. Scheepers, Christina Mühlberger, Eva Jonas

**Affiliations:** ^1^Department of Social Psychology, Salzburg University, Salzburg, Austria; ^2^Department of Social and Organizational Psychology, Leiden University, Leiden, Netherlands; ^3^Department of Social, Health, and Organizational Psychology, Utrecht University, Utrecht, Netherlands

**Keywords:** existential threat, mortality salience, imagined, arousal, emotion, physiology

## Abstract

There is mixed evidence whether reflecting on an existential threat increases negative affect and thereby elicits subjective arousal and physiological activation. Additionally, it is debated whether different existential and non-existential threats elicit different arousal responses, although systematic comparisons are lacking. The current study explored affective, subjective, and physiological arousal responses while comparing several existential threats with a non-existential threat and with a control condition. One-hundred-and-seventy-one undergraduate students were randomly allocated to one of four existential threat conditions: mortality salience (MS), freedom restriction, uncontrollability, and uncertainty; or to the non-existential threat condition: social-evaluative threat (SET); or to a control condition (TV salience). Self-reported positive/negative affect was measured before and after reflection, while subjective arousal and physiological activation (electrodermal, cardiovascular, and respiratory) were measured on a high time-scale during baseline and reflection. Results showed larger increases in self-reported negative affect, as compared to the control condition, for all existential threat conditions, while there were no differences between the control condition and threat conditions regarding positive affect, subjective arousal, skin conductance, respiratory rate, and respiratory sinus arrythmia. There were subtle differences between existential and non-existential threat conditions, most notably in affective responses. Correlations showed positive associations between negative affect and subjective arousal and between trait avoidance and subjective arousal. This study is the first to systematically compare affective, subjective, and physiological changes in arousal due to reflecting on different existential threats, as well as one non-existential threat. We showed that, as compared to a control condition, reflecting on threats has a large impact on negative affect, but no significant impact on positive affect, subjective arousal, and physiological activation.

## Introduction

A downside of the advanced human ability to think beyond the here and now is that we can anticipate threatening scenarios that may occur (Sapolsky, 2004). Imagined and anticipated threats have been defined as “cognitive representations of past stressful events or feared events in the future” (Brosschot et al., 2010, p. 407) and are the hallmark of rumination and worry (Meyer et al., 1990; Bergdahl and Bergdahl, 2002). Importantly, reflecting on and worrying about threats can elicit negative affect (Kirkegaard Thomsen, 2006), self-reported arousal (Gould et al., 2015), and physiological activation (e.g., increases in heart rate and skin conductance; Brosschot et al., 2006). For example, the mere anticipation of a social-evaluative threat e.g., giving an unexpected publicly-evaluated speech, can elicit negative affect (i.e., anxiety), increased subjective arousal, and sympathetic activation, and decreased positive affect and parasympathetic activation (Grossman and Wientjes, 2001, study 4; Dickerson and Kemeny, 2004; Gramer and Sprintschnik, 2008; Bouma et al., 2009; Childs et al., 2010; Gramer et al., 2012; Poppelaars et al., 2019).

Our ability to think beyond the here and now also allows us to fully comprehend harsh facts inherent to human existence: that death is inevitable (i.e., mortality), that we are ultimately alone in the world (i.e., isolation), that we are responsible for everything we do (i.e., freedom), and that life has no *a priori* meaning (i.e., meaning) (Yalom, 1980). There are some theoretical accounts that predict that these existential threats lead to increased negative affect and evoke subjective and physiological arousal (Tritt et al., 2012; Jonas et al., 2014). The empirical evidence for this theoretical claim, however, is limited. First, it is unclear whether reflecting on the existential given of mortality (i.e., mortality salience or MS) evokes changes in negative or positive affect; with most studies finding null effects (Lambert et al., 2014), whereas some studies only show increases in specific negative emotions such as fear/anxiety and sadness (Kastenbaum and Heflick, 2011; Lambert et al., 2014), while other studies find increases in positive affect (Rosenblatt et al., 1989; Schimel et al., 1999) or increased implicit tuning toward positive stimuli (DeWall and Baumeister, 2007; Kelley et al., 2014). Additionally, only three studies have shown increases in heart rate (HR), skin conductance (SC), and respiration rate, and decreases in parasympathetic activity in the MS condition but also in the control condition (eating or exam salience or dental pain), without significant differences between conditions (Arndt et al., 2001b, unpublished, mentioned in Rosenblatt et al., 1989; Arndt et al., 2001a, study 5; Klackl and Jonas, 2019), while another study showed lower parasympathetic activation after MS reflection as compared to a control condition (exam salience; Kosloff et al., 2008). Second, experiments on reflecting on freedom restriction have found increases in negative affect (specifically anger) (Dillard and Shen, 2005) as well as increases in HR but not in SC (Sittenthaler et al., 2015; study 3, 2016); while more experienced freedom is associated with an increased positive affect (Veenhoven, 2000). In contrast to MS and freedom restriction – which have traditionally been manipulated through reflection tasks – the physiological consequences of other existential threats, such as uncontrollability and uncertainty, have only been studied outside the field of social psychology and have typically been manipulated through physical manipulations or motivated performance tasks (i.e., performing a task, making a decision). Third, uncontrollability threat has mainly been examined using physical manipulations, especially with the anticipation of uncontrollable aversive stimuli (e.g., electric shocks, aversive tones, or photographs). This generally yields higher levels of negative affect (specifically fear/anxiety and anger), HR, and SC compared to controllable stimuli (Geer and Maisel, 1972; Maier and Seligman, 1976; Szpiler and Epstein, 1976; Miller, 1979; Thompson, 1981; Maier and Watkins, 1998; Sullivan and Lewis, 2003)^1^, although it doesn’t seem to change positive affect (Henderson et al., 2012). Fourth, regarding uncertainty, it has long been associated with feelings of anxiety (Hirsh et al., 2012; Grupe and Nitschke, 2013), but it can also increase positive affect sometimes (van den Bos, 2001; Wilson et al., 2005). Experiments examining the physiological consequences of uncertainty have been mainly focused on motivated performance situations. For example, when deciding upon an ambivalent choice, SC increased, which was mediated by self-reported uncertainty (van Harreveld et al., 2009). Similarly, during a gambling task, uncertainty (i.e., gambling on unknown probabilities) caused greater SC responses than risk (i.e., known low probability of winning) (FeldmanHall et al., 2016). To our knowledge, there have been no studies on how physiological activation changes as people reflect on isolation or meaning existential threats. To summarize, while there is considerable evidence that reflecting on certain existential threats elicits negative affect and physiological arousal, most of the existential threats have not received much attention so far, and no research has systematically compared arousal responses to reflecting on different types of existential threats.

The main goal of the current study was to compare affective/subjective arousal/physiological responses elicited by reflecting on different existential threats. Despite the variety in existential threats and the debate about whether certain existential threats elicit unique responses over others (e.g., Kirkpatrick and Navarrete, 2006; Pyszczynski et al., 2006), research comparing multiple existential threats is lacking. To our knowledge, this study is the first to systematically measure the arousal responses to different existential threats. We compared four existential threat conditions with one control condition (TV salience): MS, freedom restriction, uncontrollability, and uncertainty. Besides examining their unique effects, we also combined all existential threat conditions into a single condition and compared it with the control condition, to thereby examine the general effect of existential threat. Additionally, since it has been argued that all threat responses underlie a generalized, evolutionarily primitive, anxiety system (Tritt et al., 2012), and that those responses can be encapsulated by a general process model (Jonas et al., 2014), we also compared the existential threat conditions to a non-existential threat condition by inducing social-evaluative threat (SET). Since the motivated performance manipulation of SET is well-known to elicit negative affect and arousal (Grossman and Wientjes, 2001; Gramer and Sprintschnik, 2008; Bouma et al., 2009; Gramer et al., 2012; Poppelaars et al., 2019), we used the reflection on SET as a non-existential benchmark to compare to the existential threat conditions. Based on the theory that all threats elicit similar responses (Tritt et al., 2012; Jonas et al., 2014), we expected the non-existential and existential threats to elicit similar affective/subjective arousal/physiological responses.

Importantly, we took a more fine-grained approach to investigating arousal than previous studies by measuring arousal on a high time-scale over the whole course of threat reflection. This is a novel approach, as previous existential threat research only measured affect before and after threat reflection (Burke et al., 2010; Lambert et al., 2014) or physiological activation during threat reflection (Klackl and Jonas, 2019). Measuring both physiological and subjective arousal on a high time-scale is especially relevant since the dual process model of terror management states that thoughts of mortality are suppressed with a clear temporal sequence, i.e., (almost) immediate proximal and delayed distal defenses against death anxiety (Pyszczynski et al., 1999; Greenberg et al., 2000). Therefore, subjective arousal increases in response to MS would (almost) immediately be regulated by proximal defenses. In contrast, Lambert et al. (2014) showed that MS does in fact elicit affective responses, which can be captured by more specific measures that can distinguish between different emotions. To test these contrasting predictions, we measured physiological and subjective arousal in a high time-scale as well as self-reported affect before and after reflection and distinguished between general positive and negative affect and between several specific negative emotions. Specifically, besides using a questionnaire to measure general positive and negative affect before and after reflection, we used a content analysis of descriptions of thoughts during threat reflection to measure the number of positive and negative affect-related words, as well as three specific negative emotions: anger, fear/anxiety, and sadness. We expected to find larger increases in subjective arousal and more negative affect in the existential threat conditions as compared to the control condition, with each threat potentially evoking specific negative emotions; with MS evoking fear/anxiety and sadness (Lambert et al., 2014), freedom restriction evoking anger (Dillard and Shen, 2005), uncontrollability evoking fear/anxiety and anger (Thompson, 1981; Sullivan and Lewis, 2003), and uncertainty evoking fear/anxiety (Grupe and Nitschke, 2013). Regarding changes in positive affect, we expected MS and uncertainty to evoke no changes or increases in positive affect (Rosenblatt et al., 1989; Schimel et al., 1999; van den Bos, 2001; Hirsh et al., 2012; Lambert et al., 2014), freedom restriction to evoke decreases in positive affect (Veenhoven, 2000), and uncontrollability to evoke no changes in positive affect (Henderson et al., 2012). Additionally, we measured a large variety of indices of physiological activation during reflection: purely sympathetic activation (electrodermal using SC: Gutrecht, 1994; and cardiovascular using pre-ejection period: Cacioppo et al., 1994) or partly sympathetic/parasympathetic activation (HR and blood pressure: Cacioppo et al., 1994; respiratory rate: Grossman, 1983), and purely parasympathetic activation (respiratory sinus arrythmia: Cacioppo et al., 1994). We expected to find increases in (partly) sympathetic activation and decreases in parasympathetic activation in all existential threat conditions.

Moreover, we measured cardiac output (CO) and total peripheral resistance (TPR), which, according to the biopsychosocial (BPS) model of arousal regulation (Blascovich and Tomaka, 1996; Blascovich, 2013) can differentiate between “challenge” and “threat” motivational states. The BPS model applies specifically to motivated performance situations, proposing that when task-demands outweigh personal resources, threat emerges, and when resources approach or exceed demands, challenge emerges. Challenge is marked by increases in CO and decreases in TPR, while threat is marked by increases in TPR and decreases in CO (Blascovich and Tomaka, 1996; Blascovich, 2013). Although the reflection task we used in the current research contains some elements of motivated performance (a self-relevant goal) it is a less clear case of motivated performance compared to the tasks that are typically used in this domain (e.g., a speech task). Therefore, we anticipated that the task would yield only modest levels of task engagement (which is marked by increased HR and decreased PEP), which would in turn limit the interpretation of CO and TPR reactivity in terms of challenge and threat. However, in the case of sufficient levels of task-engagement (decreased PEP and increased HR), we explore the existence of between-condition differences in challenge and threat on the basis of CO and TPR.

Finally, since certain self-reported personality traits (e.g., high self-esteem and trait approach and low trait avoidance) have been proposed to offer anxiety/arousal-buffering effects when reflecting on threats (Greenberg et al., 1992; Leary and Baumeister, 2000; Pyszczynski et al., 2004; Schmeichel et al., 2009; Routledge et al., 2010), we also explored correlations between self-reported personality traits and arousal responses.

## Materials and Methods

### Participants

One-hundred-and-eighty-five young adults (25.8% male) participated in the experiment. Participants were recruited via social media and locally in the university area, with the requirement that they were between 18 and 35 years of age and spoke fluent German.

In total, 14 participants were excluded, either due to technical difficulties (eight participants due to recording failure, and four participants due to missing timing markers) or other reasons (one participant became unwell, and one participant revealed not to speak German fluently after all). Thus, the final sample contained 171 participants (26.3% male; 92.4% right-handed; mean age of 22.4, with a standard deviation of 3.2), with 30 participants in the MS condition, 28 participants in the freedom restriction condition, 28 participants in the uncontrollability condition, 29 participants in the uncertainty condition, 28 participants in the SET (i.e., non-existential threat) condition, and 28 participants in the control condition (TV salience). To test for general effects of threat with increased statistical power, all existential threat conditions were also *post-hoc* combined into one threat condition (*n* = 115).

Participants were compensated with course credits or €7 in cash. The experiment was approved by the ethical committee of the University of Salzburg.

### Threat Manipulation

Participants were semi-randomly allocated to the different conditions, based on the order that the participants were tested (by repeatedly cycling through a list of six conditions). Participants read a short instruction on a computer screen, asking them to think about a certain situation, as well as about the emotions their thoughts evoked and what else they thought would happen in the situation. The instructions were based on the traditional mortality salience (MS) manipulation (Rosenblatt et al., 1989), although in the current experiment participants first reflected on the situation they had to recall during a physiological recording period and only wrote down their answers afterward (to prevent movement artifacts in the physiological data). The wording of the MS instructions was subsequently adjusted to all other conditions in order to be as similar as possible to each other, only differing in the type of situation to reflect on.

Thus, in the MS condition, participants were asked to think about their own death. In the freedom restriction condition, participants were asked to think about a situation in their life where they had not been free (i.e., a situation in which someone else has forced them to either do something or to refrain from doing something). In the uncontrollability condition, participants were asked to think about the aspects of their life that make them feel powerless and that implied lacking control to influence important things in life. In the uncertainty condition, participants were asked to think about the aspects of their life that made them feel uncertain. In the SET condition, participants were asked to imagine that they were about to give an improvised socially-evaluated speech about their personality traits. Finally, in the TV salience condition (the control condition), participants were asked to think about watching television, which is a commonly-used control condition in MS research (Greenberg et al., 1994). The complete manipulation instructions can be found in the Supplementary Material.

### Procedure

**FIGURE 1:**
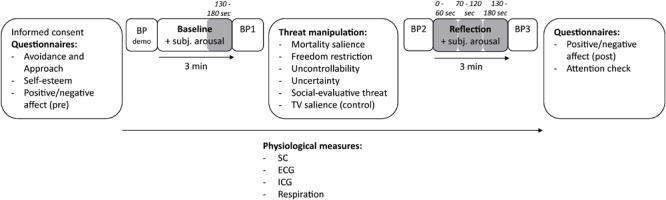
Procedure. BP, blood pressure; Subj. arousal, subjective arousal; SC, skin conductance; ECG, electrocardiography; ICG, impedance cardiography. Shaded areas indicate the physiological measurements.

An overview of the experimental procedure is displayed in Figure 1. When participants arrived at the laboratory, they provided informed consent and completed the self-reported measures of personality traits on a computer, as well as a baseline measure of self-reported positive/negative affect. Afterward, participants were led to the physiology recording area, where they were seated in front of a computer and attached to the physiological measurement equipment. Participants first practiced using the subjective arousal measure (see section “Subjective Arousal During Reflection”), and the blood pressure equipment was demonstrated. Then, a 3-min physiological baseline recording was performed, during which participants looked at a fixation cross on the screen while continuously indicating their subjective arousal level. Following this, a single blood pressure recording was taken as a baseline measurement. Subsequently, participants read the manipulation instructions on the screen and their blood pressure was again measured before they started the reflection period of 3 min. While reflecting on the instructed situation, participants looked at a fixation cross on the screen, and continuously indicated their subjective arousal levels. After the reflection period, blood pressure was measured for a third and final time. Thereafter, participants completed the positive/negative affect post-measure, as well as attention checks (see section “Attention Checks”) where they wrote down what they thought about during the reflection period. Finally, participants were debriefed. The total duration of the experiment was approximately 30 min.

### Attention Checks

To check whether participants actually reflected on the instructed situation during the 3-min reflection period, three questions were asked. The first question was: *“How well did you manage to imagine the situation?”* with a scale ranging from *1 (completely not)* to *5 (completely)*. On average, participants responded with 3.5 (between “moderate” and “well”). The second question was: *“Out of the 3 min that you had to think about the situation, how much percent of the time did you actually think about the situation?”* ranging from 0 to 100%. On average, participants responded with 69.2%. The third and final question was an open-ended question: *“Please describe shortly about what you thought about.”* These responses were used to analyze the emotional content of the thoughts during the reflection period (see section “Written Affect-Related Words”).

### Self-Reported Positive/Negative Affect After Reflection

To measure positive/negative affect, six subscales of the 12-point core affect circumplex (Yik et al., 2011) were used: activated pleasure (e.g., *enthusiastic, elated*), pleasant activation (e.g., *energetic, excited*), activation (e.g., *aroused, activated*), unpleasant activation (e.g., *frenzied, jittery*), activated displeasure (e.g., *distressed, upset*), and displeasure (e.g., *unhappy, dissatisfied*). The adjective format (translated to German) was used, with scales ranging from *not at all (1)* to *extremely (5)*. Since we were interested in valence more generally and in order to reduce the number of variables, we calculated a positive affect scale by averaging the positively valenced subscales (i.e., activated pleasure, pleasant activation) with a good internal consistency (α^pre^ = 0.85, α^post^ = 0.86), and a negative affect scale by averaging the negatively valenced subscales (i.e., unpleasant activation, activated displeasure, displeasure) with a good to excellent internal consistency (α^pre^ = 0.87, α^post^ = 0.92).

#### Written Affect-Related Words

As an exploratory additional measure of positive/negative affect, we analyzed the written responses to the open-ended attention check question (“*Please describe shortly about what you thought about*”). The German 2001 dictionary (Wolf et al., 2008) of the Linguistic Inquiry and Word Count (LIWC) 2015 software (Pennebaker et al., 2001) was used to count the percentage of affect-related words relative to all written words. Participants responded with an average of 24 words (*SD* = 23.0), consisting on average of 62% dictionary-recognized words (*SD* = 24.2). Since, ideally, these analyses would be performed with at least 50 words^2^, the results should be interpreted with caution. It should also be kept in mind that negations of emotional words – e.g., not happy – would also count as emotional words – in this case a positive affect-related word.

We analyzed positive and negative affect-related words by using all available emotional categories in the dictionary, which have been shown to be sensitive to written emotions (Kahn et al., 2007) and are commonly used (e.g., Dirkse et al., 2015). The categories are the number of words related to positive emotion (e.g., *happy, pretty, good*) and negative emotion (e.g., *hate, worthless, enemy*), as well as three specific negative emotions: anger (e.g., *hate, kill, pissed*), fear/anxiety (e.g., *nervous, afraid, tense*), and sadness (e.g., *grief, cry, sad*).

### Subjective Arousal During Reflection

To record subjective arousal on a high timescale during both baseline and reflection, we used “CARMA”: software for Continuous Affect Rating and Media Annotation (Girard, 2014). The scale, which ranged from -*100 (very calm)* to *100 (very aroused)*, was shown on the right side of a black screen with a fixation cross. Participants were instructed to indicate their current arousal level (without the instructions mentioning valence) just before the baseline and reflection period, and then to reassess this at least once every 15 s or whenever they felt that their arousal level had changed. The average subjective arousal was exported for every 10 s and averaged into 1-min bins.

### Physiological Activation During Reflection

Skin conductance, cardiovascular responses (electrocardiography and impedance cardiography), and respiration were recorded continuously using a 64-channel amplifier (TMSi, Oldenzaal, Netherlands) and Polybench 1.22 (TMSi, Oldenzaal, Netherlands) with a sampling rate of 1024 Hz. The ground electrode was situated on the non-dominant wrist.

Two software programs were used to analyze the physiological measures (except for BP): first, the ANSLAB toolbox (V2.6; Blechert et al., 2015) was used to analyze RR and RSA, the results of which were exported for the last minute of baseline, as well as for every minute of the reflection period. Secondly, the PhysioData Toolbox (V0.3.3; Sjak-Shie, 2017) was used to analyze all other measures (SC, PEP, HR, CO, TPR), the results of which were exported for the last minute of baseline, as well as for every 30 s of the reflection period, which were subsequently averaged into 1-min bins.

#### Skin Conductance (SC)

Two Ag/AgCl electrodes (MedCat, Netherlands) were attached with Velcro bands to the inside of the medial phalanges of the index and middle fingers of the participants’ non-dominant hand. The hand rested on a pillow with the palm upwards. The area bounded by the phasic signal and abscissa, divided by the epoch duration (i.e., SC, in micro siemens per second), was calculated automatically (Sjak-Shie, 2017).

#### Blood Pressure (BP)

Systolic and diastolic BP were measured from the non-dominant upper-arm using an automatic blood pressure monitor (Ecomed BU-90E, Medisana AG, Neuss, Germany), which was operated by the experimenter after the baseline period and before and after the reflection period. BP data preprocessing was performed in SPSS 24 (IBM Corp, 2016). Mean arterial pressure (MAP, in mmHg) was calculated with the formula: MAP = 2/3 diastolic blood pressure + 1/3 systolic blood pressure (Sesso et al., 2000) and used for all subsequent BP analyses.

#### Electrocardiography (ECG)

ECG was recorded using a two-lead unipolar modified chest configuration with electrodes on the right collar bone and on the lowest left rib. R-waves in the ECG were automatically detected and manually checked, and heart rate (HR, in beats per minute) was exported (Sjak-Shie, 2017).

Respiratory sinus arrhythmia (RSA) was analyzed by spectral analysis as the high frequency component of variation in inter-beat intervals (i.e., high frequency heart-rate variability) within the 0.14–0.5 Hz frequency band using fast Fourier transform with the Welch algorithm (Welch, 1967). RSA values were log-transformed (Blechert et al., 2015).

#### Respiration

Respiratory rate (RR, in cycles per minute) was assessed to account for its potential confounding influence on RSA in within-subject reactivity analyses (Grossman and Taylor, 2007). Respiration was recorded using inductive plethysmography (SleepSense, S.L.P. Inc., IL, United States) at the thoracic diaphragm. RR was calculated as 60 s divided by the continuously measured breath duration.

#### Impedance Cardiography (ICG)

ICG signals were recorded using an eight-spot pairwise electrode configuration. Pre-gelled adhesive electrodes were placed at the neck and abdomen following the guidelines by Sherwood et al. (1990). The distance between the two active electrodes was measured and noted to be later used in the calculation of cardiac output (CO).

The ICG signal was ensemble-averaged in synchrony with the ECG R-wave (Sjak-Shie, 2017). The R- and C-points of the inverted dZ/dt signal of ensemble-averaged beats were identified automatically, while the Q-, B-, and X-points were identified manually, based on offline-calculated derivatives of the ECG and ICG signal (see the Supplementary Material for scoring details).

Pre-ejection period values (PEP, in milliseconds: for which lower values represent increased ventricular contractility), mean amplitude of baseline impedance (i.e., Z0, in Ohm), the amplitude of the C- and B-points, and left ventricular ejection time (i.e., LVET) values were exported. ICG data preprocessing was performed in SPSS 24 (IBM Corp, 2016). Mean Z0 values were averaged and used as a constant in the calculation of stroke volume, for which we used the Kubicek formula (Kubicek et al., 1966). CO (in liters per minute) was calculated by multiplying HR and stroke volume. Total peripheral resistance (TPR, in dyne-seconds⋅cm^–5^) was computed by dividing MAP by CO and multiplying those values by 80 (Sherwood et al., 1990).

### Self-Reported Personality Traits

The generalized approach- and avoidance-motivated personality scales (Prentice, 2020) were used to measure whether high trait avoidance (10 items, e.g., *“When I’m faced with a decision, I am afraid to make a mistake.”*) and low trait approach (10 items, e.g., *“On the whole I am more focused on reaching profits instead of avoiding losses.”*) would increase the impact of threats. Both the trait avoidance (α = 0.87) and trait approach (α = 0.88) scales had good internal consistency.

The Rosenberg self-esteem scale (Rosenberg, 1965) was used to explore whether low self-esteem would increase the impact of threats. Besides the 10 original items (e.g., *“On the whole, I am satisfied with myself.”*), an 11th general self-esteem item was added: *“I have high self-esteem.”* The scale had acceptable consistency overall (α = 0.74).

### Statistical Analyses

Statistical analyses were performed in RStudio version 1.1.456 (RStudio Team Inc., 2018) using R version 3.5.1 (R Core Team, 2018). Both the data and the used analysis scripts are freely available via Mendeley Data (Poppelaars et al., 2020).

Reactivity (i.e., increases due to reflection) was calculated using reflection minus baseline (Seraganian et al., 1985). Specifically, for self-reported positive/negative affect, reactivity was calculated for the post-reflection minus the pre-reflection measure. For subjective arousal and physiological activity, reactivity was calculated for each minute of reflection minus the last minute of baseline. For blood pressure, reactivity was calculated for the pre- and post-reflection measurement minus the post-baseline measurement. This change score is a straightforward and easily interpretable measure of change that captures reactivity, independent of recovery.

Sixty-three percent of participants had at least one missing value due to excessive noise, sensors losing contact, or recording malfunctions. In order to retain statistical power, missing data was handled using multiple imputation with the *mice* R package (van Buuren and Groothuis-Oudshoorn, 2011). Sixty-three datasets were imputed, based on the rule of thumb that at least as many datasets need to be imputed as the percentage of incomplete cases (White et al., 2011). Missing values were imputed by predictive mean matching within 100 iterations. Each variable was predicted by other time points of the same measure (ranging between 0 and 6 variables), as well as by condition, age, sex, personality traits avoidance, approach, and self-esteem, affect-related words (six variables), positive and negative affect reactivity, subjective arousal reactivity during the third minute of reflection, and physiological reactivity during the first minute of reflection (eight variables, including BP pre-reflection, HR, SC, PEP, RR, RSA, CO, and TPR); totaling between 23 and 29 predictor variables. Reactivity variables were passively imputed; meaning that the reactivity values were not imputed directly but calculated from the imputed data instead. Plausibility of imputed variables was assessed using boxplots, strip plots, and density plots, as well as summary statistics. All subsequent analyses were performed for each of the imputed datasets and the resulting estimates were pooled according to Rubin’s rules (Rubin, 2004). There were 10 outliers in the data (based on Bonferroni-corrected Grubbs’ tests; Grubbs, 1950), which were removed in advance in order to be imputed (according to Schafer, 1997).

Before looking at reactivity, we checked whether there were any between-condition differences at baseline, using univariate ANOVAs with condition as a fixed factor. To assess whether the threats induced significant reactivity, one-sample *t*-tests against zero (i.e., no change) were used. To assess differences between conditions, two-sample *t*-tests (based on linear regressions) were used. Additionally, analyses of RSA reactivity were repeated with linear regressions and RR as a covariate, as recommended by Grossman and Taylor (2007). Finally, we ran whole-sample exploratory Pearson correlations between reactivity of positive/negative affect, subjective arousal, physiological activation, and personality traits. Non-parametric tests were used for the analyses of the written affect-related words, due to the non-normal distribution of count data; using sign tests to assess the significant presence of affect-related word categories, and Mann–Whitney *U* tests to assess condition differences.

As an effect size, Cohen’s *d* is reported (except for the sign tests for significant word counts). Additionally, Bayes factors (with default uninformed priors) using the *BayesFactor* R package (Morey and Rouder, 2018) were calculated (except for the non-normally distributed written affect-related words) to complement the frequentist statistics and clarify whether non-significant findings should be interpreted as inconclusive or in support of the null hypothesis (Kass and Raftery, 1995). Our interpretation of Bayes factors and their cutoffs was based on Kass and Raftery (1995), specifically the cutoff for substantial evidence for the null hypothesis (*BF* < 0.3125). Multiple comparison correction was applied for all analyses using false-discovery rate (FDR; Benjamini and Yekutieli, 2001). FDR-corrected *p*-values smaller than 0.05 were deemed significant. Both uncorrected and FDR-corrected *p*-values are reported for transparency.

#### Power Analysis

A *post-hoc* statistical power calculation conducted with G^∗^Power (Faul et al., 2007) showed that the final sample size offered 80% power to detect changes in reactivity variables with medium effect sizes (between *d* = 0.47 and *d* = 0.48) for individual conditions and small effect sizes (*d* = 0.23) for the existential threat composite. Additionally, the final sample size offered 80% power to detect differences with the control condition with medium-large effect sizes (between *d* = 0.75 and *d* = 0.77) for individual conditions and with medium effect sizes (*d* = 0.60) for the existential threat composite. Finally, the complete sample size offered 80% power to detect correlations with small effect sizes (*d* = 0.19). Additional use of Bayesian model comparison offered the distinction between non-significant results due to lack of power or due to support for the null hypothesis.

## Results

ANOVAs indicated no significant effect of condition or combined existential threat conditions on any of the baseline values of the dependent variables, uncorrected *p*’s > 0.096, FDR-corrected *p*’s > 0.961.

### Positive/Negative Affect After Reflection

**FIGURE 2:**
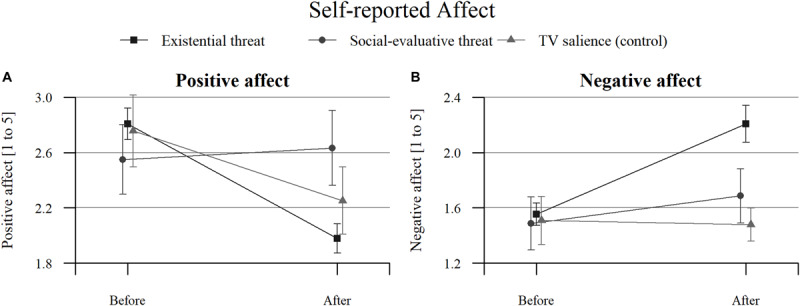
Self-reported affect before and after reflection within three aggregate conditions (existential threat composite, social-evaluative threat, and TV salience) for: **(A)** positive effect, and **(B)** negative effect. Error bars represent 95% confidence intervals.

**FIGURE 3:**
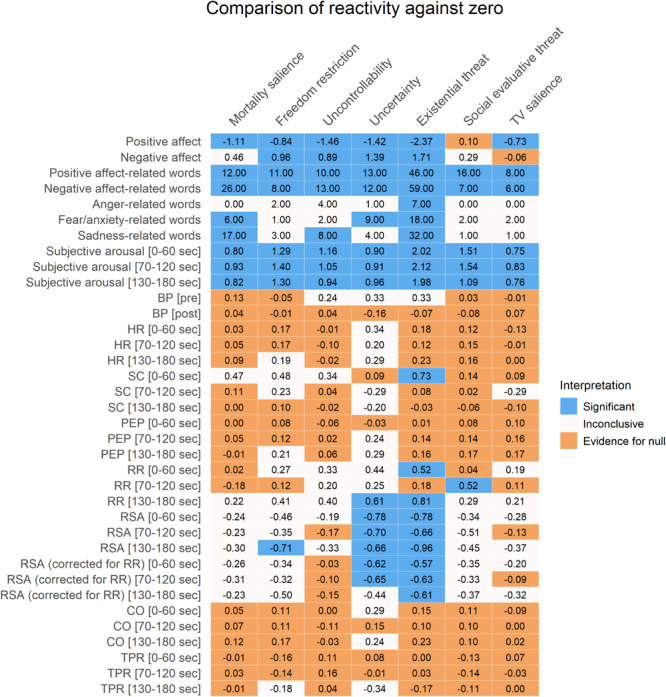
Results of one sample *t*-tests of reactivity comparing separate conditions against zero. Cohen’s *d* effect sizes are displayed for all variables except for word-related variables, which show S values instead. Colors represent significant *p*-values after FDR-correction (in blue), substantial Bayesian evidence for the null hypothesis (in orange), or inconclusive results (in off-white). The existential threat column is a composite of the first four columns. SC, skin conductance; HR, heart rate; PEP, pre-ejection period; BP, blood pressure; RSA, respiratory sinus arrhythmia; RR, respiratory rate; CO, cardiac output; TPR, total peripheral resistance.

**FIGURE 4:**
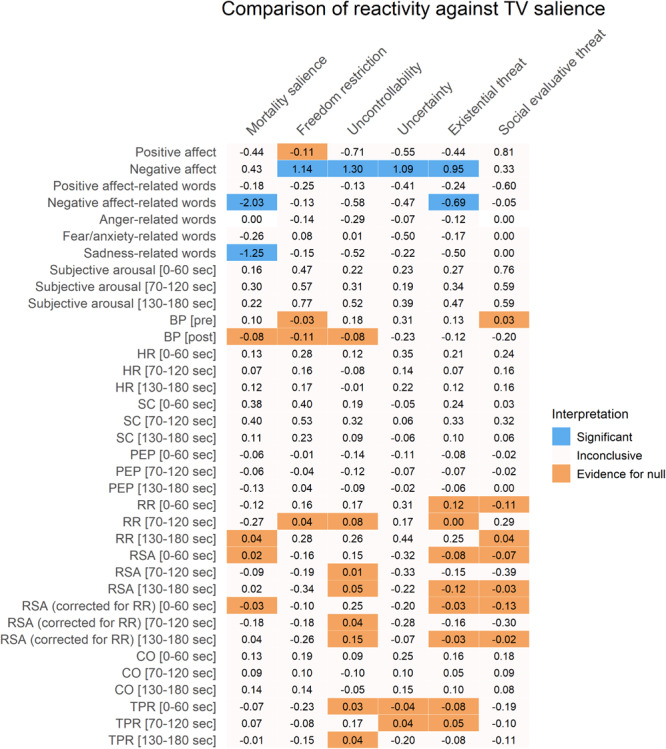
Results of two sample *t*-tests of reactivity comparing separate conditions against the control condition (TV salience). Cohen’s *d* effect sizes are displayed. Colors represent significant *p*-values after FDR-correction (in blue), substantial Bayesian evidence for the null hypothesis (in orange), or inconclusive results (in off-white). The existential threat column is a composite of the first four columns. SC, skin conductance; HR, heart rate; PEP, pre-ejection period; BP, blood pressure; RSA, respiratory sinus arrhythmia; RR, respiratory rate; CO, cardiac output; TPR, total peripheral resistance.

**FIGURE 5:**
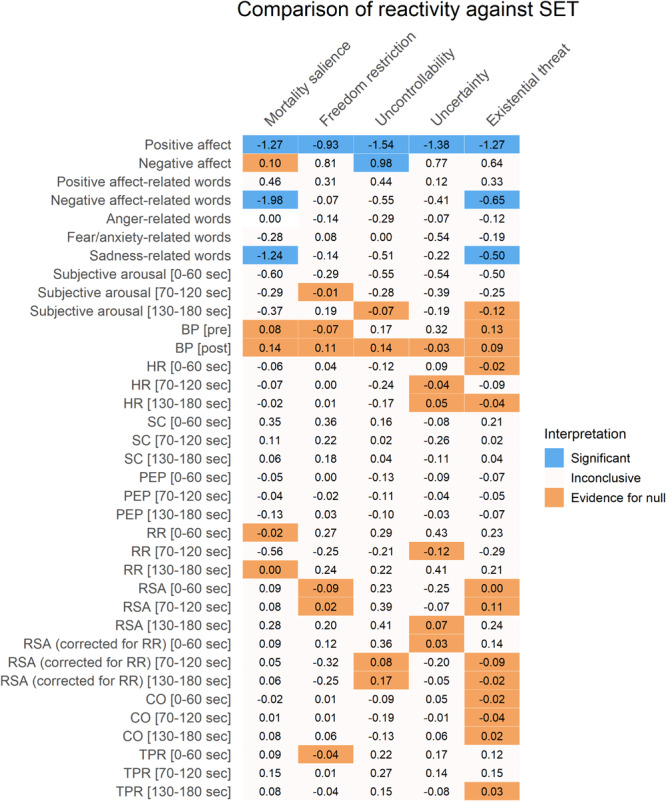
Results of two sample *t*-tests of reactivity comparing existential threat conditions against the social-evaluative threat condition. Cohen’s *d* effect sizes are displayed. Colors represent significant *p*-values after FDR-correction (in blue), substantial Bayesian evidence for the null hypothesis (in orange), or inconclusive results (in off-white). The existential threat column is a composite of the first four columns. SC, skin conductance; HR, heart rate; PEP, pre-ejection period; BP, blood pressure; RSA, respiratory sinus arrhythmia; RR, respiratory rate; CO, cardiac output; TPR, total peripheral resistance.

Plots for the three aggregate conditions (existential threat composite, social-evaluative threat, and TV salience) can be found in Figure 2 and plots for each separate condition can be found in Supplementary Figure S1. An overview of the positive and negative affect results can be found in Figures 3–5, with detailed results in Supplementary Tables S1–S3.

Self-reported positive affect decreased from before to after the manipulation in all conditions except for the SET condition, which did not change. This decrease was not more pronounced in the threat conditions than in the TV salience condition: Only the freedom restriction condition did not differ from the TV salience condition, while all other conditions had inconclusive results. All separate existential threat conditions (including the existential threat composite) showed larger decreases in positive affect than the SET condition.

Self-reported negative affect increased in the freedom restriction, uncontrollability, and uncertainty conditions, as well as for the existential threat composite, and these increases exceeded those seen in the TV salience condition. In the TV salience condition itself, negative affect did not change, while the MS and SET conditions had inconclusive results. The uncontrollability condition showed larger increases in negative affect than the SET condition, while the MS condition did not differ from the SET condition.

#### Written Affect-Related Words

**FIGURE 6:**
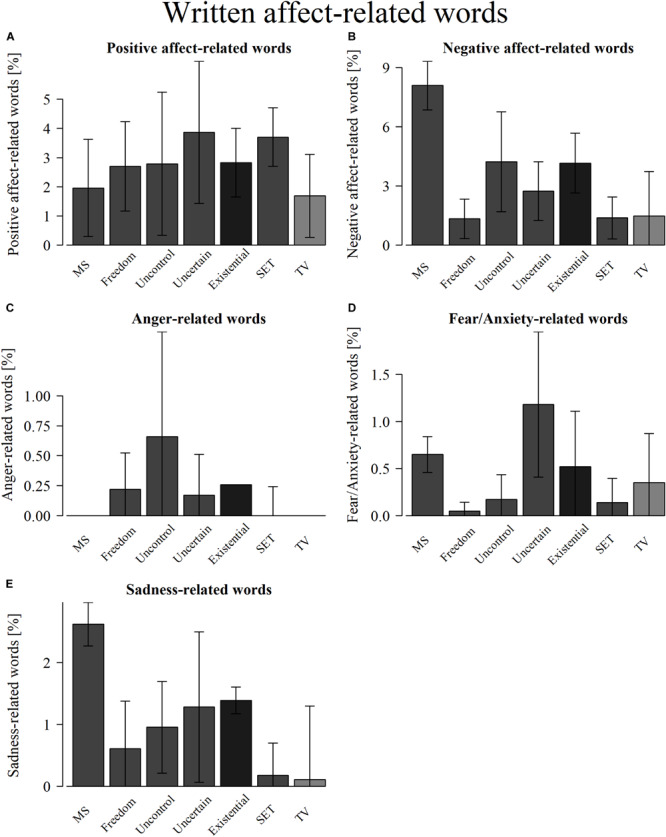
Written affect-related words within all conditions (mortality salience, freedom restriction, uncontrollability, uncertainty, existential threat composite, social-evaluative threat, and TV salience) for: **(A)** positive affect-related words, **(B)** negative affect-related words, **(C)** anger-related words, **(D)** fear/anxiety-related words, and **(E)** sadness-related words. Error bars represent 95% confidence intervals.

An overview of the written affect-related word results can be found in Figures 3–5, with detailed results in Supplementary Tables S1–S3. Plots of the results can be found in Figure 6.

Results showed that participants in all conditions reported positive and negative affect-related thoughts, which significantly differed from zero, though only the MS condition and combined existential threat conditions reported more negative affect-related thoughts than the control condition or the SET condition.

For anger-related thoughts, only the combined existential threat conditions significantly reported anger, although this could not be compared to the control or SET conditions due to a lack of observations.

For fear/anxiety-related thoughts, the MS and uncertainty conditions and the existential threat composite significantly reported fear/anxiety, though none reported more fear/anxiety than the control or SET conditions.

For sadness-related thoughts, the MS and uncontrollability conditions and the existential threat composite significantly reported sadness, though only the MS condition and the existential threat composite reported significantly more sadness than the SET condition, and only the MS condition also reported more sadness than the control condition.

### Subjective Arousal During Reflection

**FIGURE 7:**
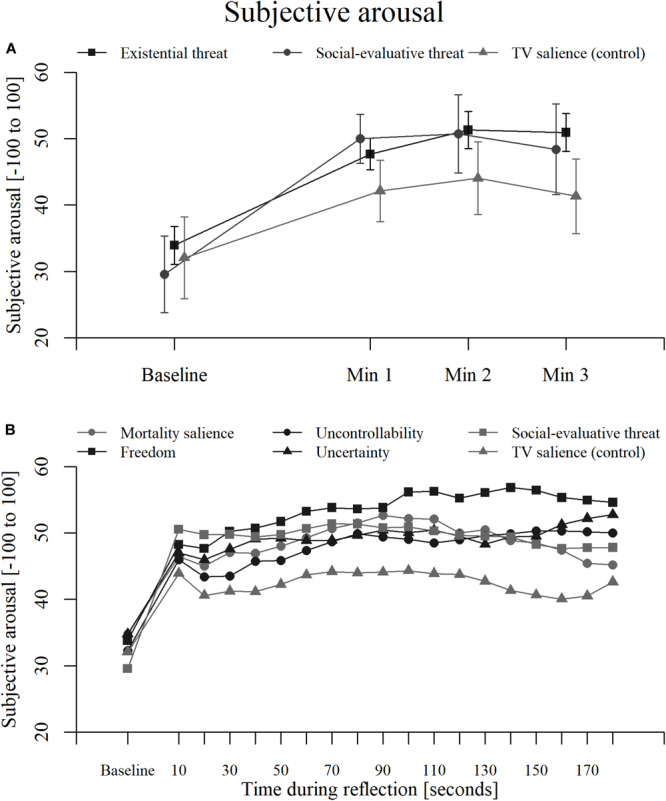
Subjective arousal over time within **(A)** three aggregate conditions (existential threat composite, social-evaluative threat, and TV salience) for every 60 s of reflection, and **(B)** all separate conditions (mortality salience, freedom restriction, uncontrollability, uncertainty, social-evaluative threat, and TV salience) for every 10 s of reflection. Error bars represent 95% confidence intervals.

An overview of the subjective arousal results can be found in Figures 3–5, with detailed results in Supplementary Tables S1–S3. A plot for the three aggregate conditions (existential threat composite, social-evaluative threat, and TV salience) can be found in Figure 7A and a plot for each separate condition can be found in Supplementary Figure S2. Additionally, Figure 7B illustrates the subjective arousal responses for every 10 s of reflection for each separate condition.

Subjective arousal increased in all conditions for each minute of the reflection. However, the magnitude of these increases was not significantly higher for the threatening conditions than for the TV salience condition. There was substantial Bayesian evidence for no differences between the SET condition and freedom condition during minute two and between the SET condition and the uncontrollability condition and all combined existential threat conditions during minute three.

### Physiological Activation During Reflection

**FIGURE 8:**
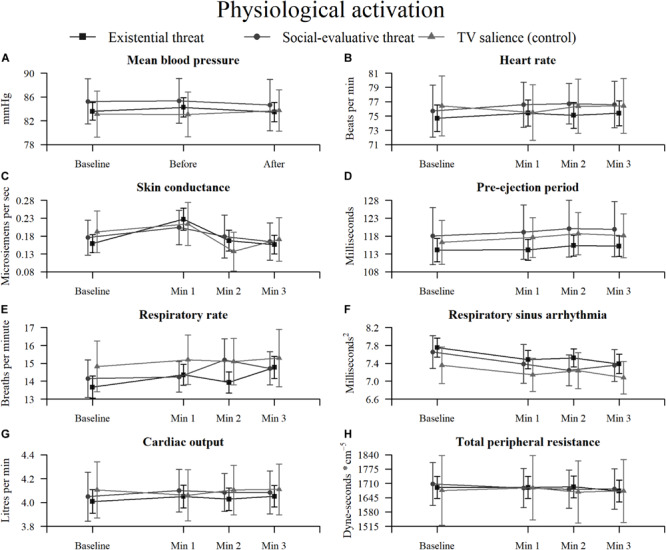
Physiological activation over time within three aggregate conditions (existential threat composite, social-evaluative threat, and TV salience) for: **(A)** mean blood pressure, **(B)** heart rate, **(C)** skin conductance, **(D)** pre-ejection period, **(E)** respiratory rate, **(F)** respiratory sinus arrhythmia, **(G)** cardiac output, and **(H)** total peripheral resistance. Error bars represent 95% confidence intervals.

An overview of the physiological activation results can be found in Figures 3–5, with detailed results in Supplementary Tables S1–S3. Plots for the three aggregate conditions (existential threat composite, social-evaluative threat, and TV salience) can be found in Figure 8 and plots for each separate condition can be found in Supplementary Figure S3.

Results showed significant increases in SC (i.e., sympathetic electrodermal activity) in the existential threat composite during the first minute of reflection, although there was inconclusive evidence for larger increases in SC compared to the control or SET condition. Additionally, all combined existential threat conditions showed significant increases in RR (i.e., partly-sympathetic respiratory activity) during the first and last minute of reflection, as well as for the uncertainty condition in the last minute and for the SET condition during the second minute, though again there was either inconclusive evidence or substantial evidence for no differences compared to the control or SET condition. Finally, RSA (i.e., parasympathetic cardiovascular activity) showed significant decreases during the first minute (uncertainty condition and the existential threat composite), second minute (uncertainty condition and the existential threat composite), and the third minute of reflection (freedom restriction and uncertainty conditions, and the existential threat composite); which did not change when correcting for RR reactivity for the first and second minute, although the effect disappeared for the freedom restriction and uncertainty conditions for the third minute; again there was either inconclusive evidence or substantial evidence for no differences in RSA reactivity compared to the control or SET condition.

All other physiological measures (BP, HR, PEP, CO, TPR) largely showed substantial evidence for no changes and no differences between individual conditions compared to the control condition or the SET condition, with some inconclusive results.

### Correlations

**FIGURE 9:**
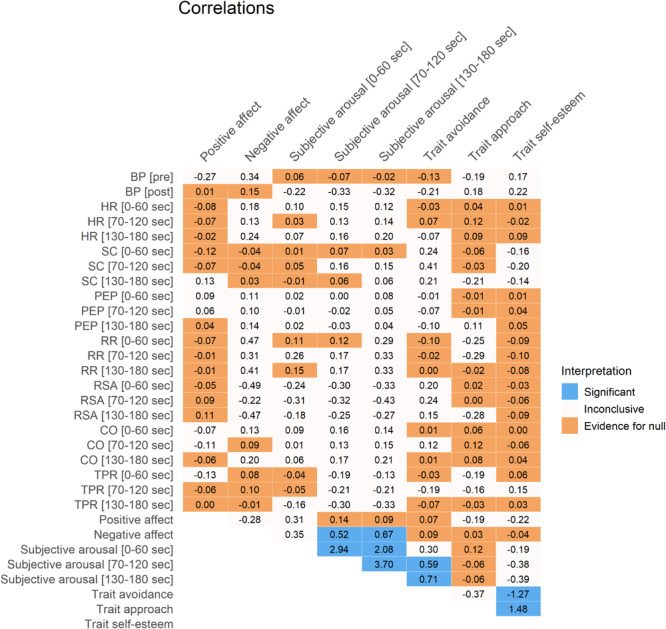
Correlations between reactivity of self-reported affect, subjective arousal, physiological activation, and personality traits. Cohen’s *d* effect sizes are displayed. Colors represent significant *p*-values after FDR-correction (in blue), substantial Bayesian evidence for the null hypothesis (in orange), or inconclusive results (in off-white). SC, skin conductance; HR, heart rate; PEP, pre-ejection period; BP, blood pressure; RSA, respiratory sinus arrhythmia; RR, respiratory rate; CO, cardiac output; TPR, total peripheral resistance.

**FIGURE 10:**
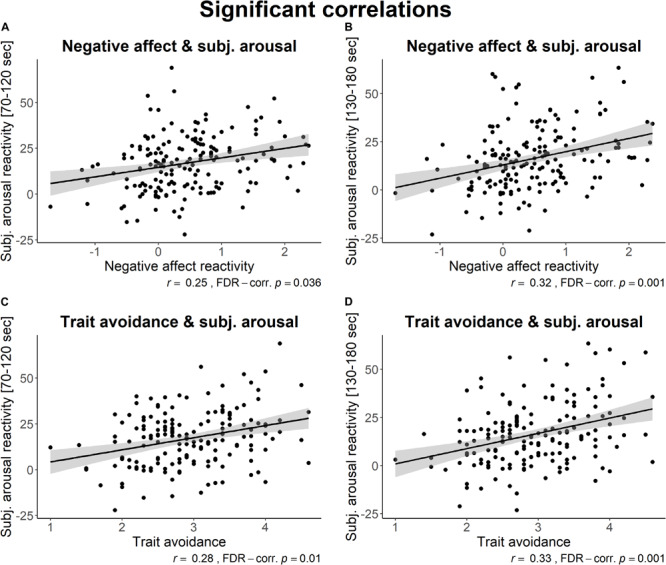
Scatterplots of significant correlations for **(A)** negative affect and subjective arousal (min 2), **(B)** negative affect and subjective arousal (min 3), **(C)** trait avoidance and subjective arousal (min 2), and **(D)** trait avoidance and subjective arousal (min 3). Pearson correlation coefficients and FDR-corrected *p*-values are displayed. The first imputed dataset is used for illustrative purposes. The 95% confidence interval is displayed in the shaded gray area, and the linear regression line is displayed in black.

An overview of the correlation results between reactivity of self-reported affect, subjective arousal, physiological activation, and personality traits can be found in Figure 9, with detailed results in Supplementary Table S4. Plots of the interesting significant correlations can be found in Figure 10.

Results showed interesting medium-strength positive associations between negative affect reactivity and subjective arousal reactivity (for minutes two and three), as well as between trait avoidance and subjective arousal reactivity (for minutes two and three). Additionally, as could be expected, there were associations with large effect sizes between the three different minutes of subjective arousal, and between the trait self-esteem and traits avoidance and approach (though not between traits avoidance and approach itself).

Notably, there was either inconclusive evidence or substantial evidence for no associations between physiological activity and affect/subjective arousal/traits.

## Discussion

The aim of the present study was to investigate whether reflecting on (existential) threats elicits changes in self-reported affect, subjective arousal, and physiological activation, and whether these changes differ from a control condition (TV salience). As hypothesized, results indicated that reflection elicited increases in negative affect, subjective arousal, (partly) sympathetic activation (SC, RR) and decreases in positive affect and parasympathetic activation (RSA). However, while the existential threat conditions evoked larger increases in negative affect than the control condition did, the threat and control conditions did not differ in terms of changes in positive affect, subjective arousal, or physiological activation. Therefore, we conclude that reflecting on threats in particular has a large impact on negative affect, but no significant impact on positive affect, subjective arousal, or physiological activation.

Our arousal results are in line with previous research showing that reflecting on existential threats evokes large increases in negative affect (Kastenbaum and Heflick, 2011; Lambert et al., 2014), as well as small increases in electrodermal sympathetic activation (SC) and partly sympathetic respiratory activation (RR), and small decreases in parasympathetic activity (e.g., Miller, 1979; Rosenblatt et al., 1989; Arndt et al., 2001a; Kosloff et al., 2008; Sittenthaler et al., 2016; Klackl and Jonas, 2019), with no differences between the threat and control condition as found in most research manipulating MS through reflection (Miller, 1979; Rosenblatt et al., 1989; Arndt et al., 2001a; Klackl and Jonas, 2019) but unlike the common physical manipulations of uncontrollability (Geer and Maisel, 1972; Szpiler and Epstein, 1976; Miller, 1979) or the performance tasks manipulating uncertainty (van Harreveld et al., 2009; FeldmanHall et al., 2016). Since our task was a reflection task and we modeled all manipulations onto the structure of a typical MS manipulation, it makes sense that our results are more in line with previous MS research.

The current study extends the literature on affect and physiological activation by also measuring subjective arousal during reflection on a high time-scale. The subjective arousal responses showed large increases during threat reflection, although these did not differ from the control condition. These results demonstrate an absence of arousal suppression due to proximal defenses, as would be predicted by the dual defense model of terror management (Pyszczynski et al., 1999; Greenberg et al., 2000). Interestingly, although both the subjective arousal and affective measures were self-reported and the physiological activation measures were objective, the subjective arousal results largely mirrored the physiological responses instead of the affective responses. It can be questioned why our results showed divergent evidence for changes in negative affect on the one hand, and subjective and physiological arousal measures on the other hand. Although it has long been assumed that affective and physiological responses are associated (Lindsley, 1951; Darwin, 1965), it is in fact a common finding within stress research that affective responses are not associated with physiological responses (Campbell and Ehlert, 2012). This might be due to measuring the subjective arousal and physiological activation on a similar time-scale during the reflection task and the affective responses only before and after the task (Barrett, 1997; Hellhammer and Schubert, 2012), or due to the complex associations that exist between stress response systems, depending – among other things – on threat type, threat interpretation, and on the baseline state, gender, menstrual cycle, and personality of the participant (Poppelaars et al., 2019). Therefore, we conclude that it is not completely unexpected to find large changes in negative affect due to threat reflection but only small changes in subjective arousal or physiological activation.

The MS condition differed from the other threat conditions in terms of its sensitivity to the two measures of self-reported affect. Participants in the MS condition showed inconclusive changes in negative affect after reflection, with no significantly-larger increases in negative affect than in the control condition. This lack of affective responses has also been found in previous MS studies and forms the basis of the “affect-free claim” (Lambert et al., 2014). However, this is in contrast with the results of the content analysis of the written words describing the thoughts during the reflection period. By analyzing these written thoughts, it was found that the MS condition evoked significantly more negative emotions – specifically sadness – than the control condition. Similarly, mainly sadness, as well as anxiety, were found in a previous study’s descriptions of thoughts during MS (Kastenbaum and Heflick, 2011). On the one hand, this contrast might be due to the specificity of the measures; while negative affect was very generally defined, written affect-related words could distinguish between three specific negative emotions. According to Lambert et al. (2014), the affect-free claim has resulted from a lack of specificity of measured emotions, whereas the specific measurement of the emotions fear and sadness do increase notably during MS. On the other hand, this contrast might be due to the timing of the affect measurements: whereas negative/positive affect was measured right after the reflection period, affect-related words were only measured at the end of the experiment (i.e., distally), when death thoughts might already be outside of focal attention but are still easily accessible according to the dual process model of terror management (Pyszczynski et al., 1999; Greenberg et al., 2000). Indeed, it has previously been found that negative affect after MS is only increased when measured distally; which is thought to be caused by emotion suppression via proximal defenses (Routledge et al., 2010). However, since the act of writing about thoughts during the reflection brings death-related thoughts back into focal attention, this possible explanation can be considered refuted. Thus, it seems likely that the lack of larger increases in negative affect as compared to the control condition are not due to timing influences, but rather due to emotion specificity, since the MS condition did indeed write more sadness-related words than the control condition.

Considering associations between changes in affect, subjective arousal, physiological activation, and personality traits, we found no associations with physiological reactivity. Our results did show medium-strength associations between higher trait avoidance and larger increases in subjective arousal, which in turn were related to increased negative affect. This is consistent with several studies showing that those who use more avoidance coping tend to feel more stressed (Holahan and Moos, 1986; Holahan et al., 2005). Therefore, further research on threat reflection with vulnerable populations would be interesting, such as those with avoidant personality disorder and those that heavily rely on avoidant emotion regulation.

To answer the main question of the study – whether reflecting on (existential) threat evokes negative affect, subjective arousal, and physiological activation – our results indicated that brief episodes of reflecting on existential givens do elicit large increases in negative affect, even when compared to a control condition. Threat reflection also evoked small decreases in positive affect, increases in subjective arousal, and changes in physiological activation, but these changes were not higher than those seen in a non-threatening control condition.

Another aim of the current study was to compare the arousal responses elicited by reflecting on four different existential threats: mortality salience (MS), freedom restriction, uncontrollability, and uncertainty; and one non-existential threat: social-evaluative threat (SET). Although all threat conditions showed similar patterns overall, there were subtle differences between the existential threat conditions and the non-existential threat condition, most notably in terms of affect. Specifically, regarding negative affect: whereas all existential threats (except for MS) showed increases in negative affect and the MS condition reported more negative affect- and sadness-related thoughts, SET showed inconclusive changes in negative affect and specific affect-related words; with the uncontrollability condition even showing larger increases in negative affect as compared to the SET condition and the MS condition reporting more negative affect- and sadness-related thoughts than the SET condition. Regarding positive affect: whereas all existential threats showed decreases in positive affect, SET showed no change in positive affect, with all existential threats showing larger decreases in positive affect as compared to SET. Overall, we posit that the theory that all threats (i.e., both existential and non-existential) cause similar arousal responses (Tritt et al., 2012; Jonas et al., 2014) was partially supported; because even though there were subtle differences in affective responses between reflecting on different threats, all threat conditions did show similar large increases in subjective arousal as well as medium-strength changes in the same directions for physiological activation (i.e., increases in skin conductance and respiratory rate and decreases in respiratory sinus arrhythmia), with those changes all not being significantly different from those in the control condition. Thus, people experience similar levels of arousal after reflecting on both existential and non-existential threats, as proposed by the general process model of threat and defense (Jonas et al., 2014), but there are some differences between existential and non-existential threats on a descriptive level. Indeed, as the current study is the first study to systematically compare different (existential) threats, more research is needed, and replication of our results is warranted in order to confirm our conclusion.

The current study also has several limitations. First, the sample size of the individual conditions was small (ranging from 28 to 30 participants), restricting our statistical power and thus our confidence in the results. Specifically, a *post-hoc* power analysis showed that we only had sufficient statistical power to detect arousal changes with medium effect sizes and condition differences with medium-large effect sizes, which might not be sufficient for the typically-small affective changes caused by MS (e.g., Burke et al., 2010). This was noticeable in the large number of non-significant results that showed inconclusive Bayesian evidence; and thus, these inconclusive results should be further investigated in future well-powered studies. However, with the use of the existential threat composite (115 participants), our statistical power increased and was able to detect arousal changes with small effect sizes and condition differences with medium effect sizes, which also showed a smaller number of inconclusive results. Secondly, we only used self-reported measures of positive/negative affect, and hence, these measures could be biased by socially-desirable responding and consistency seeking (Paulhus and Vazire, 2007). Indeed, it is possible that the increase in negative affect after existential threat can be explained by the experimental demands. Additionally, the increase in negative affect-related words is to be expected when writing about an objectively-negative situation such as an existential threat. However, this alternative explanation cannot explain the different timelines of each condition of our more fine-grained subjective arousal measure, which was also self-reported-although this measure was underpowered and thus did not find differences between conditions. Third, because of the relatively passive nature of the reflection task, we anticipated only modest levels of task-engagement. However, since we found no evidence for sufficient task-engagement based on cardiovascular measures, this precluded a further interpretation of cardiovascular reactivity in terms of motivational states of challenge and threat. Although the current task carries some aspects of motivated performance (i.e., a self-relevant goal), the task was not strong enough to elicit clear task-engagement. In future research, the task could be slightly adapted, for example by including the expectation that one has to verbally report on the situation later (see Mendes et al., 2002), allowing a threat/challenge analysis of different types of existential threats. Fourth and final, we grouped freedom restriction as an existential threat, although it can be debated whether this can actually be viewed as a threat to one’s meaningful existence. On the one hand, although the four existential concerns of Yalom (1980) do include freedom, this is defined more in terms of responsibility. In reactance theory, on the other hand, freedom is always defined with regard to specific situations, so Brehm and Brehm (1981) did not consider freedom to be an existential threat. Others, however, do classify freedom restriction as an existential threat (Koole et al., 2006), which we also did for simplicity.

## Conclusion

In conclusion, the current study was the first to systematically compare affective, subjective, and physiological changes in arousal due to reflecting on different existential threats, as well as one non-existential threat. We showed that, as compared to a control condition, reflecting on threats has a large impact on negative affect, but no significant impact on positive affect, subjective arousal, and physiological activation. All threats caused similar responses overall, with the only notable differences between existential and non-existential threats regarding affective responses.

## Data Availability Statement

All datasets generated for this study are included in Poppelaars et al. (2020), as well as in the Supplementary Material.

## Ethics Statement

The studies involving human participants were reviewed and approved by Ethics Committee of the University of Salzburg. The participants provided their written informed consent to participate in this study.

## Author Contributions

EP: methodology, software, formal analysis, data curation, writing – original draft, writing – review and editing, visualization, and project administration. JK and EJ: conceptualization, methodology, writing – review and editing, supervision, and funding acquisition. DS: software, resources, writing – review and editing, and supervision. CM: methodology, writing – review and editing, and project administration.

## Conflict of Interest

The authors declare that the research was conducted in the absence of any commercial or financial relationships that could be construed as a potential conflict of interest.

## Footnotes

1 However, extreme uncontrollability can also lead to *reduced* SC because of learned helplessness (Seligman, 1972; Gatchel and Proctor, 1976).

2 liwc.wpengine.com/how-it-works
