# Supplementary material for: Reflecting on Existential Threats Elicits Self-Reported Negative Affect but No Physiological Arousal
Source: Front Psychol. 2020 May 29;11:962. doi: 10.3389/fpsyg.2020.00962 (PMC7273972; doi:10.3389/fpsyg.2020.00962)
Supplement: Supplementary file 1 [file Table_1.docx]

***Supplementary Material***

**Materials and Methods**

**Manipulation instructions**

The complete manipulation instructions per condition in German are provided underneath, as well as a non-validated English translation for illustrative purposes.

*1. Mortality salience (English)*

Please think about your own mortality.

- Please think about the emotions that are evoked by the thoughts about your own death.
- Think about what would happen to you when you die and when you are dead.

*1. Mortality salience (German)*

Denken Sie bitte über Ihre eigene Sterblichkeit nach.

- Bitte denken Sie an die Emotionen, die der Gedanke an Ihren eigenen Tod bei Ihnen hervorruft.
- Denken Sie auch darüber nach, was Ihrer Meinung nach mit Ihnen passiert wenn Sie sterben und wenn Sie dann tot sind.

*2. Freedom restriction (English)*

Please think about a situation in your life in which you are not free, i.e. situations in which someone else has forced you to either do something or to refrain from doing something.

- Please think about the emotions that are evoked by your thoughts about freedom restriction.
- Think about what would happen to you when your freedom is restricted.

*2. Freedom restriction (German)*

Denken Sie bitte an Situationen in Ihrem Leben, in denen Sie unfrei sind, d.h. Situationen, in denen Sie jemand dazu gezwungen hat, etwas zu tun oder etwas zu unterlassen.

- Bitte denken Sie an die Emotionen, die der Gedanke an diese Unfreiheit bei Ihnen hervorruft.
- Denken Sie bitte darüber nach, was mit Ihnen passiert, wenn Sie unfrei sind.

*3. Uncontrollability (English)*

Please think about the aspects in your life that make you feel powerless and lacking control to influence the important things in your life.

- Please think about the emotions that are evoked by your thoughts about powerlessness.
- Think about what would happen to you when you are powerless and lack control.

*3. Uncontrollability (German)*

Denken Sie bitte an Aspekte in Ihrem Leben, die Ihnen das Gefühl eigener Machtlosigkeit und mangelnden Einflusses auf die wichtigen Dinge Ihres Lebens vermitteln.

- Bitte denken Sie an die Emotionen, die der Gedanke an diese Machtlosigkeit bei Ihnen hervorruft.
- Denken Sie bitte darüber nach, was mit Ihnen passiert, wenn Sie machtlos sind und keinen Einfluss haben.

*4. Uncertainty (English)*

Please think about the aspects in your life that make you feel uncertain. You can feel uncertain about yourself, about your life, or about your future.

- Please think about the emotions that are evoked by your thoughts about uncertainty.
- Think about what would happen to you when you are uncertain.

*4. Uncertainty (German)*

Denken Sie bitte an die Aspekte in Ihrem Leben, die Sie unsicher machen. Unsicher über Sie selbst, über Ihr Leben und Ihre Zukunft.

- Bitte denken Sie an die Emotionen, die der Gedanke an diese Unsicherheit bei Ihnen hervorruft.
- Denken Sie bitte darüber nach, was Ihrer Meinung nach mit Ihnen passiert wenn Sie unsicher sind.

*5. Social-evaluative threat (English)*

Please imagine that you would have to give a speech about yourself in three minutes. This speech should last about five minutes. You will talk about your good and bad qualities. Your task is to structure your speech, so that is it a logically flowing story; to tell it in a funny and enthusiastic way; and to speak clearly. Imagine that you will give this speech in front of a camera beside you, and that the speech will be recorded. The quality of your recorded speech will be evaluated by eight people at a later time point.

- Please think about the emotions that are evoked by your thoughts about the speech and the subsequent evaluation.
- Think about what would happen to you in that situation.

*5. Social-evaluative threat (German)*

Stellen Sie sich bitte vor, dass sie in drei Minuten eine Rede über Ihre Person halten werden. Diese wird ca. fünf Minuten dauern. In der Rede soll es um Ihre persönlichen guten und schlechten Eigenschaften gehen. Sie bekommen die Aufgabe, Ihre Rede gut zu strukturieren, sodass es eine logisch aufgebaute Geschichte wird, lustig und enthusiastisch zu erzählen und klar zu sprechen. Stellen Sie sich vor, dass Sie die Rede vor der Kamera neben Ihnen halten und dass die Rede aufgezeichnet wird. Später wird die Qualität Ihrer Rede von acht Personen bewertet.

- Bitte denken Sie an die Emotionen, die der Gedanke an die Rede und anschließende Bewertung bei Ihnen hervorruft.
- Denken Sie auch darüber nach, was dabei mit Ihnen passiert.

*6. TV Salience (English)*

Please think about watching TV.

- Please think about the emotions that are evoked by your thoughts about watching TV.
- Think about what would happen to you when you watch TV.

*6. TV Salience (German)*

Denken Sie bitte über Fernsehen nach.

- Bitte denken Sie an die Emotionen, die der Gedanke an Fernsehen bei Ihnen hervorruft.
- Denken Sie auch darüber nach, was Ihrer Meinung nach mit Ihnen passiert wenn Sie fernsehen.

**Physiological Activation**

**Impedance cardiography (ICG).** The ICG data were scored in the PhysioData toolbox (Sjak-Shie, 2017) according to standard guidelines (Sherwood et al., 1990; Debski et al., 1993; Seery et al., 2016). The scoring of the Q-point was based on R-onset, as identified by the last negative-to-positive crossing of the 2^nd^ derivative before the R-peak. The scoring of the X-point was based on the first negative-to-positive crossing of the 2^nd^ derivative after the C-point. The scoring of the B-point was preferably based on the last negative-to-positive crossing of the 2^nd^ derivative before the C-point. When this was not possible, the last negative-to-positive crossing of the 3^rd^ derivative before the C-point was used instead. When that was also not possible, the peak of the 3^rd^ derivative before the C-point was used.

**Results**

Plots of positive/negative affect per each condition can be found in Figure S1. A plot of subjective arousal per each condition can be found in Figure S2. Plots of physiological activation per each condition can be found in Figure S3.

Results of one sample t-tests of reactivity comparing separate conditions against zero can be found in Table S1. Results of two sample t-tests of reactivity comparing separate conditions against the control condition (TV salience) can be found in Table S2. Results of two sample t-tests of reactivity comparing existential threat conditions against the social-evaluative threat condition can be found in Table S3. Results of the correlations between reactivity of affect, subjective arousal, physiological activation, and personality traits can be found in Table S4. Interpretations of these results are discussion in the main manuscript.


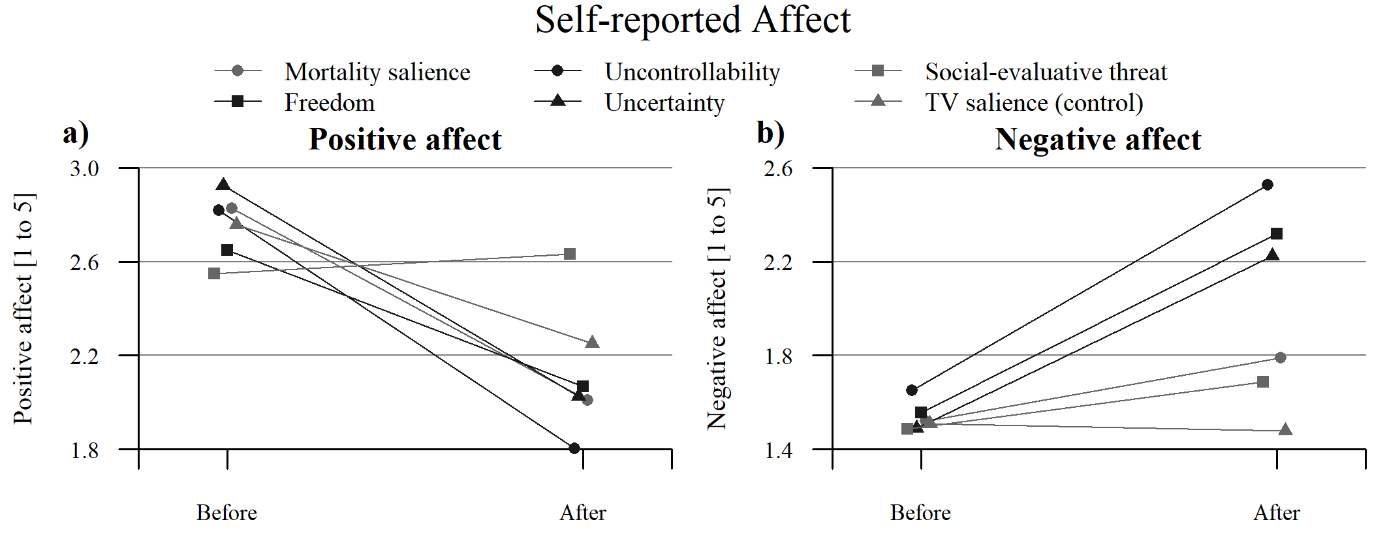


Figure S1. Affect before and after reflection within all separate conditions (mortality salience, freedom restriction, uncontrollability, uncertainty, social-evaluative threat, and tv salience) for: a) positive affect, and b) negative affect.


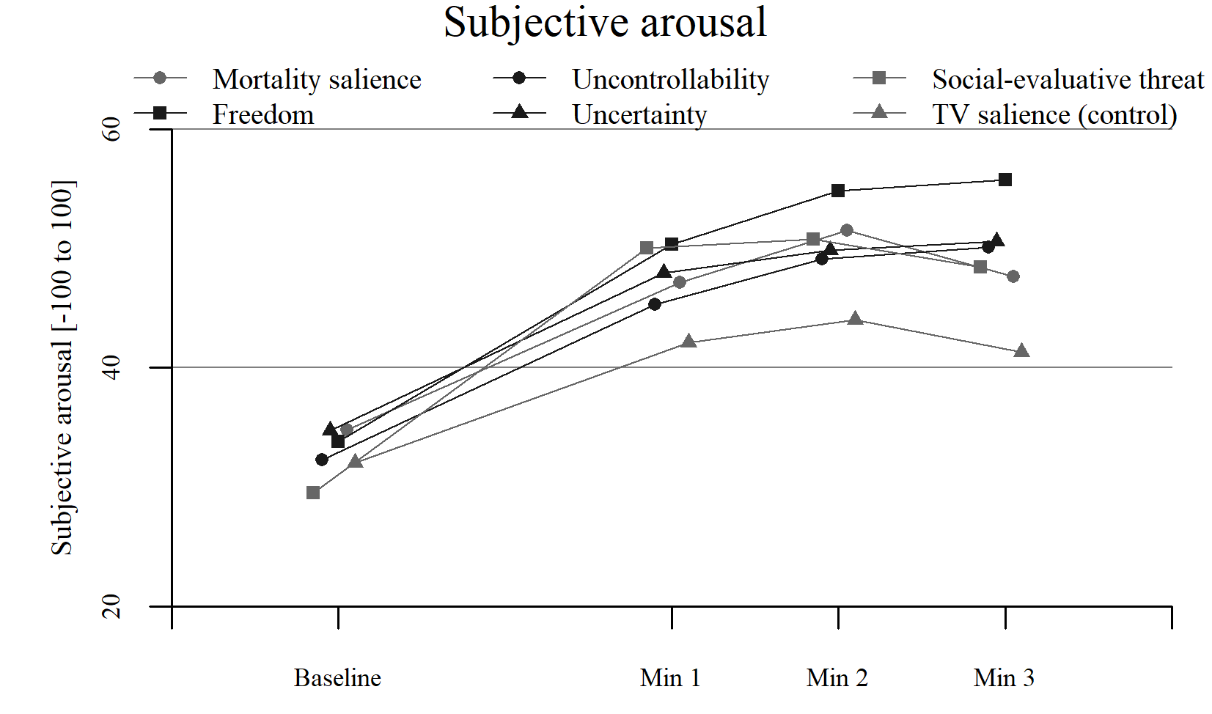


Figure S2. Subjective arousal responses over time within all separate conditions (mortality salience, freedom restriction, uncontrollability, uncertainty, social-evaluative threat, and tv salience).


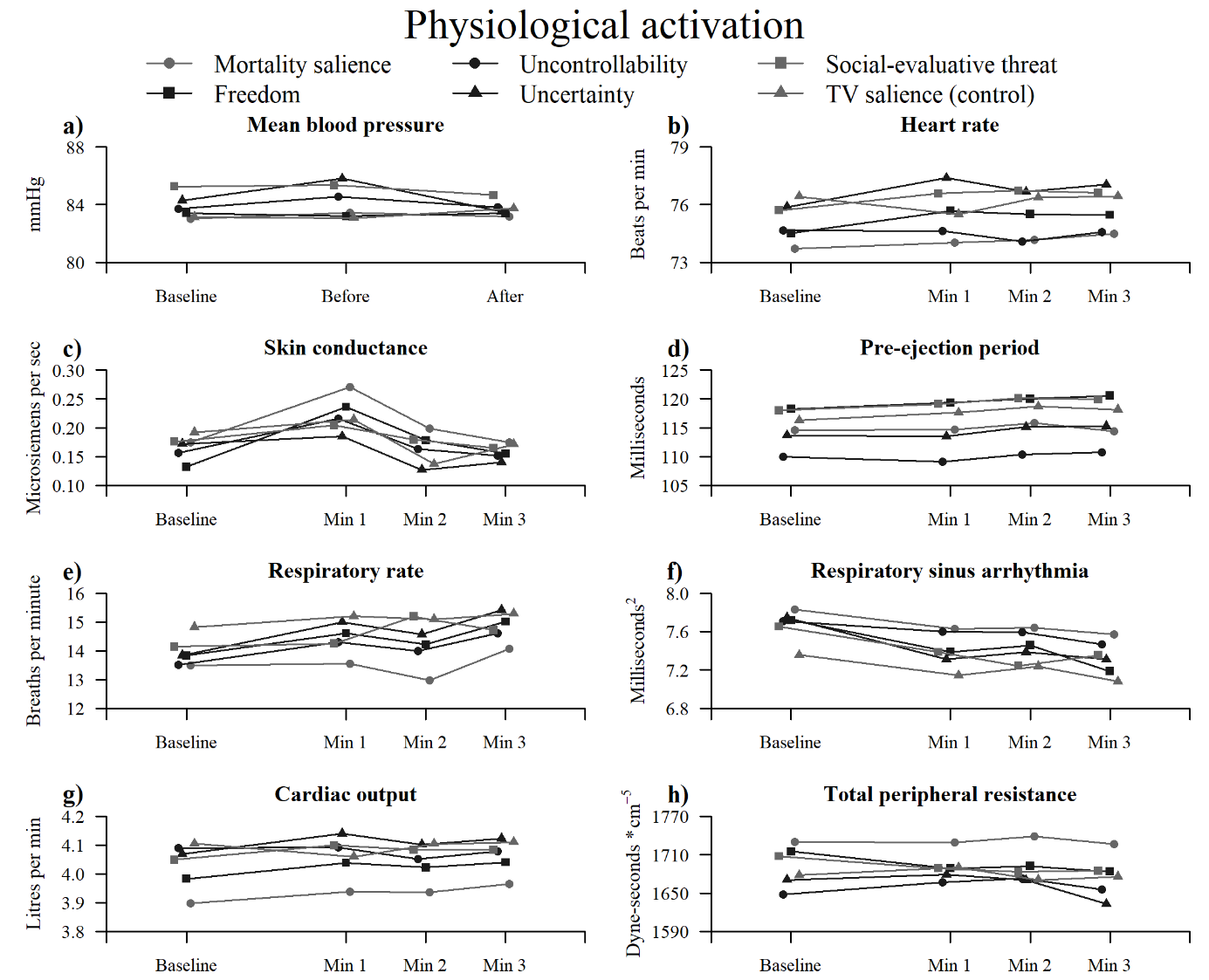


Figure S3. Physiological activation over time within all separate conditions (mortality salience, freedom restriction, uncontrollability, uncertainty, social-evaluative threat, and tv salience) for: a) mean blood pressure, b) heart rate, c) skin conductance, d) pre-ejection period, e) respiratory rate, f) respiratory sinus arrhythmia, g) cardiac output, and h) total peripheral resistance.

Table S1. Results of one sample *t*-tests of reactivity comparing separate conditions against zero.

|  | Mortality salience | Freedom restriction | Uncontroll-ability | Uncertainty | Existential threat composite | Social-evaluative threat | TV salience |
| --- | --- | --- | --- | --- | --- | --- | --- |
| Positive affect | ***t*(27) = -5.97, uncorr. *p* < .001, FDR-corr. *p* < .001, BF = 10517.27, *d* = 1.11** | ***t*(25) = -4.50, uncorr. *p* < .001, FDR-corr. *p* = .001, BF = 229.31, *d* = 0.84** | ***t*(25) = -7.82, uncorr. *p* < .001, FDR-corr. *p* < .001, BF = 655409.41, *d* = 1.46** | ***t*(26) = -7.60, uncorr. *p* < .001, FDR-corr. *p* < .001, BF = 492014.44, *d* = 1.42** | ***t*(112) = -12.69, uncorr. *p* < .001, FDR-corr. *p* < .001, BF = 2.778306e+20, *d* = 2.37** | ***t*(25) = 0.53, uncorr. *p* = .603, FDR-corr. *p* = .835, BF = 0.23, *d* = 0.10** | ***t*(24) = -3.94, uncorr. *p* = .001, FDR-corr. *p* = .004, BF = 59.87, *d* = 0.73** |
| Negative affect | *t*(27) = 2.47, uncorr. *p* = .020, FDR-corr. *p* = .086, BF = 2.54, *d* = 0.46 | ***t*(25) = 5.15, uncorr. *p* < .001, FDR-corr. *p* < .001, BF = 1101.83, *d* = 0.96** | ***t*(25) = 4.75, uncorr. *p* < .001, FDR-corr. *p* = .001, BF = 418.84, *d* = 0.89** | ***t*(26) = 7.43, uncorr. *p* < .001, FDR-corr. *p* < .001, BF = 327449.65, *d* = 1.39** | ***t*(112) = 9.15, uncorr. *p* < .001, FDR-corr. *p* < .001, BF = 2.492418e+12, *d* = 1.71** | *t*(25) = 1.54, uncorr. *p* = .136, FDR-corr. *p* = .358, BF = 0.57, *d* = 0.29 | ***t*(24) = -0.32, uncorr. *p* = .749, FDR-corr. *p* = .926, BF = 0.21, *d* = 0.06** |
| Positive affect-related words | **S = 12, uncorr. *p* < .001, FDR-corr. *p* = .001** | **S = 11, uncorr. *p* < .001, FDR-corr. *p* = .001** | **S = 10, uncorr. *p* = .001, FDR-corr. *p* = .003** | **S = 13, uncorr. *p* < .001, FDR-corr. *p* < .001** | **S = 46, uncorr. *p* < .001, FDR-corr. *p* < .001** | **S = 16, uncorr. *p* < .001, FDR-corr. *p* < .001** | **S = 8, uncorr. *p* = .004, FDR-corr. *p* = .008** |
| Negative affect-related words | **S = 26, uncorr. *p* < .001, FDR-corr. *p* < .001** | **S = 8, uncorr. *p* = .004, FDR-corr. *p* = .008** | **S = 13, uncorr. *p* < .001, FDR-corr. *p* < .001** | **S = 12, uncorr. *p* < .001, FDR-corr. *p* = .001** | **S = 59, uncorr. *p* < .001, FDR-corr. *p* < .001** | **S = 7, uncorr. *p* = .008, FDR-corr. *p* = .014** | **S = 6, uncorr. *p* = .016, FDR-corr. *p* = .026** |
| Anger-related words | S = 0, uncorr. *p* = 1.000, FDR-corr. *p* = 1.000 | S = 2, uncorr. *p* = .250, FDR-corr. *p* = .312 | S = 4, uncorr. *p* = .062, FDR-corr. *p* = .095 | S = 1, uncorr. *p* = .500, FDR-corr. *p* = .547 | **S = 7, uncorr. *p* = .008, FDR-corr. *p* = .014** | S = 0, uncorr. *p* = 1.000, FDR-corr. *p* = 1.000 | S = 0, uncorr. *p* = 1.000, FDR-corr. *p* = 1.000 |
| Fear/anxiety-related words | **S = 6, uncorr. *p* = .016, FDR-corr. *p* = .026** | S = 1, uncorr. *p* = .500, FDR-corr. *p* = .547 | S = 2, uncorr. *p* = .250, FDR-corr. *p* = .312 | **S = 9, uncorr. *p* = .002, FDR-corr. *p* = .005** | **S = 18, uncorr. *p* < .001, FDR-corr. *p* < .001** | S = 2, uncorr. *p* = .250, FDR-corr. *p* = .312 | S = 2, uncorr. *p* = .250, FDR-corr. *p* = .312 |
| Sadness-related words | **S = 17, uncorr. *p* < .001, FDR-corr. *p* < .001** | S = 3, uncorr. *p* = .125, FDR-corr. *p* = .182 | **S = 8, uncorr. *p* = .004, FDR-corr. *p* = .008** | S = 4, uncorr. *p* = .062, FDR-corr. *p* = .095 | **S = 32, uncorr. *p* < .001, FDR-corr. *p* < .001** | S = 1, uncorr. *p* = .500, FDR-corr. *p* = .547 | S = 1, uncorr. *p* = .500, FDR-corr. *p* = .547 |
| Subjective arousal [0-60 sec] | ***t*(26) = 4.30, uncorr. *p* < .001, FDR-corr. *p* = .001, BF = 154.18, *d* = 0.80** | ***t*(24) = 6.91, uncorr. *p* < .001, FDR-corr. *p* < .001, BF = 78665.00, *d* = 1.29** | ***t*(24) = 6.21, uncorr. *p* < .001, FDR-corr. *p* < .001, BF = 14827.29, *d* = 1.16** | ***t*(25) = 4.83, uncorr. *p* < .001, FDR-corr. *p* = .001, BF = 548.82, *d* = 0.90** | ***t*(108) = 10.82, uncorr. *p* < .001, FDR-corr. *p* < .001, BF = 1.557804e+16, *d* = 2.02** | ***t*(25) = 8.09, uncorr. *p* < .001, FDR-corr. *p* < .001, BF = 1206066.91, *d* = 1.51** | ***t*(23) = 4.01, uncorr. *p* = .001, FDR-corr. *p* = .004, BF = 71.24, *d* = 0.75** |
| Subjective arousal [70-120 sec] | ***t*(26) = 4.98, uncorr. *p* < .001, FDR-corr. *p* < .001, BF = 863.04, *d* = 0.93** | ***t*(24) = 7.52, uncorr. *p* < .001, FDR-corr. *p* < .001, BF = 331685.60, *d* = 1.40** | ***t*(25) = 5.66, uncorr. *p* < .001, FDR-corr. *p* < .001, BF = 3847.72, *d* = 1.05** | ***t*(25) = 4.91, uncorr. *p* < .001, FDR-corr. *p* < .001, BF = 660.83, *d* = 0.91** | ***t*(106) = 11.34, uncorr. *p* < .001, FDR-corr. *p* < .001, BF = 2.383841e+17, *d* = 2.12** | ***t*(25) = 8.23, uncorr. *p* < .001, FDR-corr. *p* < .001, BF = 1654248.03, *d* = 1.54** | ***t*(23) = 4.45, uncorr. *p* < .001, FDR-corr. *p* = .001, BF = 202.00, *d* = 0.83** |
| Subjective arousal [130-180 sec] | ***t*(26) = 4.39, uncorr. *p* < .001, FDR-corr. *p* = .001, BF = 195.50, *d* = 0.82** | ***t*(24) = 6.97, uncorr. *p* < .001, FDR-corr. *p* < .001, BF = 90693.69, *d* = 1.30** | ***t*(25) = 5.03, uncorr. *p* < .001, FDR-corr. *p* < .001, BF = 822.13, *d* = 0.94** | ***t*(24) = 5.17, uncorr. *p* < .001, FDR-corr. *p* < .001, BF = 1273.45, *d* = 0.96** | ***t*(105) = 10.62, uncorr. *p* < .001, FDR-corr. *p* < .001, BF = 5.301463e+15, *d* = 1.98** | ***t*(25) = 5.86, uncorr. *p* < .001, FDR-corr. *p* < .001, BF = 6377.83, *d* = 1.09** | ***t*(22) = 4.06, uncorr. *p* < .001, FDR-corr. *p* = .003, BF = 79.63, *d* = 0.76** |
| BP [pre] | ***t*(27) = 0.70, uncorr. *p* = .490, FDR-corr. *p* = .783, BF = 0.24, *d* = 0.13** | ***t*(25) = -0.29, uncorr. *p* = .777, FDR-corr. *p* = .948, BF = 0.21, *d* = 0.05** | *t*(25) = 1.29, uncorr. *p* = .210, FDR-corr. *p* = .501, BF = 0.42, *d* = 0.24 | *t*(26) = 1.77, uncorr. *p* = .089, FDR-corr. *p* = .275, BF = 0.78, *d* = 0.33 | *t*(112) = 1.79, uncorr. *p* = .076, FDR-corr. *p* = .256, BF = 0.49, *d* = 0.33 | ***t*(25) = 0.14, uncorr. *p* = .890, FDR-corr. *p* = .992, BF = 0.20, *d* = 0.03** | ***t*(20) = -0.04, uncorr. *p* = .967, FDR-corr. *p* = .997, BF = 0.20, *d* = 0.01** |
| BP [post] | ***t*(27) = 0.21, uncorr. *p* = .834, FDR-corr. *p* = .983, BF = 0.20, *d* = 0.04** | ***t*(25) = -0.03, uncorr. *p* = .974, FDR-corr. *p* = .997, BF = 0.20, *d* = 0.01** | ***t*(25) = 0.22, uncorr. *p* = .828, FDR-corr. *p* = .982, BF = 0.20, *d* = 0.04** | ***t*(26) = -0.86, uncorr. *p* = .398, FDR-corr. *p* = .710, BF = 0.28, *d* = 0.16** | ***t*(112) = -0.36, uncorr. *p* = .720, FDR-corr. *p* = .908, BF = 0.11, *d* = 0.07** | ***t*(25) = -0.43, uncorr. *p* = .670, FDR-corr. *p* = .880, BF = 0.22, *d* = 0.08** | ***t*(21) = 0.38, uncorr. *p* = .709, FDR-corr. *p* = .900, BF = 0.21, *d* = 0.07** |
| HR [0-60 sec] | ***t*(14) = 0.15, uncorr. *p* = .883, FDR-corr. *p* = .992, BF = 0.20, *d* = 0.03** | ***t*(15) = 0.92, uncorr. *p* = .374, FDR-corr. *p* = .708, BF = 0.29, *d* = 0.17** | ***t*(13) = -0.03, uncorr. *p* = .976, FDR-corr. *p* = .997, BF = 0.20, *d* = 0.01** | *t*(26) = 1.84, uncorr. *p* = .078, FDR-corr. *p* = .260, BF = 0.86, *d* = 0.34 | ***t*(52) = 0.95, uncorr. *p* = .349, FDR-corr. *p* = .682, BF = 0.16, *d* = 0.18** | ***t*(14) = 0.63, uncorr. *p* = .538, FDR-corr. *p* = .821, BF = 0.24, *d* = 0.12** | ***t*(15) = -0.68, uncorr. *p* = .507, FDR-corr. *p* = .803, BF = 0.25, *d* = 0.13** |
| HR [70-120 sec] | ***t*(15) = 0.25, uncorr. *p* = .806, FDR-corr. *p* = .967, BF = 0.20, *d* = 0.05** | ***t*(16) = 0.90, uncorr. *p* = .380, FDR-corr. *p* = .710, BF = 0.29, *d* = 0.17** | ***t*(14) = -0.53, uncorr. *p* = .601, FDR-corr. *p* = .835, BF = 0.23, *d* = 0.10** | *t*(26) = 1.09, uncorr. *p* = .285, FDR-corr. *p* = .608, BF = 0.34, *d* = 0.20 | ***t*(59) = 0.64, uncorr. *p* = .525, FDR-corr. *p* = .808, BF = 0.13, *d* = 0.12** | ***t*(15) = 0.79, uncorr. *p* = .439, FDR-corr. *p* = .744, BF = 0.27, *d* = 0.15** | ***t*(16) = -0.04, uncorr. *p* = .966, FDR-corr. *p* = .997, BF = 0.20, *d* = 0.01** |
| HR [130-180 sec] | ***t*(16) = 0.49, uncorr. *p* = .628, FDR-corr. *p* = .843, BF = 0.22, *d* = 0.09** | *t*(16) = 1.03, uncorr. *p* = .320, FDR-corr. *p* = .654, BF = 0.32, *d* = 0.19 | ***t*(15) = -0.08, uncorr. *p* = .934, FDR-corr. *p* = .997, BF = 0.20, *d* = 0.02** | *t*(26) = 1.58, uncorr. *p* = .127, FDR-corr. *p* = .358, BF = 0.60, *d* = 0.29 | ***t*(60) = 1.23, uncorr. *p* = .222, FDR-corr. *p* = .519, BF = 0.22, *d* = 0.23** | ***t*(16) = 0.86, uncorr. *p* = .402, FDR-corr. *p* = .710, BF = 0.28, *d* = 0.16** | ***t*(16) < 0.01, uncorr. *p* = .997, FDR-corr. *p* = .997, BF = 0.20, *d* < 0.01** |
| SC [0-60 sec] | *t*(17) = 2.52, uncorr. *p* = .022, FDR-corr. *p* = .089, BF = 2.82, *d* = 0.47 | *t*(11) = 2.59, uncorr. *p* = .025, FDR-corr. *p* = .100, BF = 3.25, *d* = 0.48 | *t*(15) = 1.83, uncorr. *p* = .087, FDR-corr. *p* = .274, BF = 0.87, *d* = 0.34 | ***t*(17) = 0.47, uncorr. *p* = .645, FDR-corr. *p* = .853, BF = 0.22, *d* = 0.09** | ***t*(60) = 3.92, uncorr. *p* < .001, FDR-corr. *p* = .001, BF = 119.89, *d* = 0.73** | ***t*(15) = 0.74, uncorr. *p* = .468, FDR-corr. *p* = .769, BF = 0.26, *d* = 0.14** | ***t*(19) = 0.50, uncorr. *p* = .622, FDR-corr. *p* = .843, BF = 0.22, *d* = 0.09** |
| SC [70-120 sec] | ***t*(17) = 0.58, uncorr. *p* = .570, FDR-corr. *p* = .835, BF = 0.23, *d* = 0.11** | *t*(13) = 1.22, uncorr. *p* = .243, FDR-corr. *p* = .543, BF = 0.39, *d* = 0.23 | ***t*(15) = 0.19, uncorr. *p* = .853, FDR-corr. *p* = .989, BF = 0.20, *d* = 0.04** | *t*(15) = -1.57, uncorr. *p* = .137, FDR-corr. *p* = .358, BF = 0.59, *d* = 0.29 | ***t*(50) = 0.41, uncorr. *p* = .685, FDR-corr. *p* = .880, BF = 0.11, *d* = 0.08** | ***t*(14) = 0.09, uncorr. *p* = .929, FDR-corr. *p* = .997, BF = 0.20, *d* = 0.02** | *t*(16) = -1.55, uncorr. *p* = .141, FDR-corr. *p* = .365, BF = 0.58, *d* = 0.29 |
| SC [130-180 sec] | ***t*(14) < 0.01, uncorr. *p* = .997, FDR-corr. *p* = .997, BF = 0.19, *d* < 0.01** | ***t*(13) = 0.55, uncorr. *p* = .592, FDR-corr. *p* = .835, BF = 0.23, *d* = 0.10** | ***t*(12) = -0.11, uncorr. *p* = .911, FDR-corr. *p* = .997, BF = 0.20, *d* = 0.02** | *t*(15) = -1.05, uncorr. *p* = .312, FDR-corr. *p* = .651, BF = 0.32, *d* = 0.20 | ***t*(52) = -0.17, uncorr. *p* = .862, FDR-corr. *p* = .989, BF = 0.10, *d* = 0.03** | ***t*(15) = -0.33, uncorr. *p* = .746, FDR-corr. *p* = .926, BF = 0.21, *d* = 0.06** | ***t*(17) = -0.54, uncorr. *p* = .594, FDR-corr. *p* = .835, BF = 0.23, *d* = 0.10** |
| PEP [0-60 sec] | ***t*(12) = 0.02, uncorr. *p* = .984, FDR-corr. *p* = .997, BF = 0.19, *d* < 0.01** | ***t*(13) = 0.42, uncorr. *p* = .680, FDR-corr. *p* = .880, BF = 0.22, *d* = 0.08** | ***t*(12) = -0.32, uncorr. *p* = .753, FDR-corr. *p* = .926, BF = 0.21, *d* = 0.06** | ***t*(22) = -0.14, uncorr. *p* = .892, FDR-corr. *p* = .992, BF = 0.20, *d* = 0.03** | ***t*(44) = 0.03, uncorr. *p* = .976, FDR-corr. *p* = .997, BF = 0.10, *d* = 0.01** | ***t*(13) = 0.42, uncorr. *p* = .684, FDR-corr. *p* = .880, BF = 0.22, *d* = 0.08** | ***t*(12) = 0.53, uncorr. *p* = .607, FDR-corr. *p* = .835, BF = 0.23, *d* = 0.10** |
| PEP [70-120 sec] | ***t*(13) = 0.25, uncorr. *p* = .807, FDR-corr. *p* = .967, BF = 0.20, *d* = 0.05** | ***t*(14) = 0.65, uncorr. *p* = .525, FDR-corr. *p* = .808, BF = 0.24, *d* = 0.12** | ***t*(13) = 0.12, uncorr. *p* = .908, FDR-corr. *p* = .997, BF = 0.20, *d* = 0.02** | *t*(21) = 1.27, uncorr. *p* = .216, FDR-corr. *p* = .510, BF = 0.41, *d* = 0.24 | ***t*(51) = 0.75, uncorr. *p* = .458, FDR-corr. *p* = .768, BF = 0.14, *d* = 0.14** | ***t*(13) = 0.73, uncorr. *p* = .478, FDR-corr. *p* = .779, BF = 0.26, *d* = 0.14** | ***t*(12) = 0.87, uncorr. *p* = .402, FDR-corr. *p* = .710, BF = 0.28, *d* = 0.16** |
| PEP [130-180 sec] | ***t*(12) = -0.04, uncorr. *p* = .966, FDR-corr. *p* = .997, BF = 0.19, *d* = 0.01** | *t*(13) = 1.14, uncorr. *p* = .275, FDR-corr. *p* = .597, BF = 0.36, *d* = 0.21 | ***t*(13) = 0.32, uncorr. *p* = .755, FDR-corr. *p* = .926, BF = 0.21, *d* = 0.06** | *t*(22) = 1.56, uncorr. *p* = .133, FDR-corr. *p* = .358, BF = 0.58, *d* = 0.29 | ***t*(48) = 0.86, uncorr. *p* = .395, FDR-corr. *p* = .710, BF = 0.15, *d* = 0.16** | ***t*(13) = 0.89, uncorr. *p* = .389, FDR-corr. *p* = .710, BF = 0.29, *d* = 0.17** | ***t*(13) = 0.92, uncorr. *p* = .375, FDR-corr. *p* = .708, BF = 0.29, *d* = 0.17** |
| RR [0-60 sec] | ***t*(20) = 0.10, uncorr. *p* = .921, FDR-corr. *p* = .997, BF = 0.20, *d* = 0.02** | *t*(24) = 1.43, uncorr. *p* = .165, FDR-corr. *p* = .416, BF = 0.50, *d* = 0.27 | *t*(25) = 1.75, uncorr. *p* = .093, FDR-corr. *p* = .280, BF = 0.77, *d* = 0.33 | *t*(25) = 2.36, uncorr. *p* = .026, FDR-corr. *p* = .103, BF = 2.07, *d* = 0.44 | ***t*(100) = 2.80, uncorr. *p* = .006, FDR-corr. *p* = .028, BF = 4.23, *d* = 0.52** | ***t*(23) = 0.20, uncorr. *p* = .840, FDR-corr. *p* = .985, BF = 0.20, *d* = 0.04** | *t*(21) = 1.00, uncorr. *p* = .327, FDR-corr. *p* = .663, BF = 0.32, *d* = 0.19 |
| RR [70-120 sec] | ***t*(20) = -0.96, uncorr. *p* = .349, FDR-corr. *p* = .682, BF = 0.30, *d* = 0.18** | ***t*(24) = 0.65, uncorr. *p* = .523, FDR-corr. *p* = .808, BF = 0.24, *d* = 0.12** | *t*(25) = 1.08, uncorr. *p* = .291, FDR-corr. *p* = .614, BF = 0.34, *d* = 0.20 | *t*(25) = 1.34, uncorr. *p* = .192, FDR-corr. *p* = .475, BF = 0.44, *d* = 0.25 | ***t*(100) = 0.98, uncorr. *p* = .332, FDR-corr. *p* = .667, BF = 0.16, *d* = 0.18** | ***t*(21) = 2.80, uncorr. *p* = .011, FDR-corr. *p* = .048, BF = 4.91, *d* = 0.52** | ***t*(21) = 0.57, uncorr. *p* = .575, FDR-corr. *p* = .835, BF = 0.23, *d* = 0.11** |
| RR [130-180 sec] | *t*(20) = 1.16, uncorr. *p* = .260, FDR-corr. *p* = .575, BF = 0.36, *d* = 0.22 | *t*(24) = 2.22, uncorr. *p* = .036, FDR-corr. *p* = .136, BF = 1.63, *d* = 0.41 | *t*(25) = 2.14, uncorr. *p* = .043, FDR-corr. *p* = .157, BF = 1.41, *d* = 0.40 | ***t*(25) = 3.27, uncorr. *p* = .003, FDR-corr. *p* = .015, BF = 13.46, *d* = 0.61** | ***t*(99) = 4.34, uncorr. *p* < .001, FDR-corr. *p* < .001, BF = 512.40, *d* = 0.81** | *t*(21) = 1.55, uncorr. *p* = .136, FDR-corr. *p* = .358, BF = 0.58, *d* = 0.29 | *t*(21) = 1.15, uncorr. *p* = .263, FDR-corr. *p* = .576, BF = 0.36, *d* = 0.21 |
| RSA [0-60 sec] | *t*(17) = -1.30, uncorr. *p* = .210, FDR-corr. *p* = .501, BF = 0.42, *d* = 0.24 | *t*(22) = -2.49, uncorr. *p* = .021, FDR-corr. *p* = .088, BF = 2.65, *d* = 0.46 | *t*(25) = -1.02, uncorr. *p* = .316, FDR-corr. *p* = .654, BF = 0.32, *d* = 0.19 | ***t*(25) = -4.18, uncorr. *p* < .001, FDR-corr. *p* = .002, BF = 109.96, *d* = 0.78** | ***t*(87) = -4.21, uncorr. *p* < .001, FDR-corr. *p* = .001, BF = 322.41, *d* = 0.78** | *t*(23) = -1.83, uncorr. *p* = .080, FDR-corr. *p* = .261, BF = 0.86, *d* = 0.34 | *t*(22) = -1.51, uncorr. *p* = .146, FDR-corr. *p* = .371, BF = 0.55, *d* = 0.28 |
| RSA [70-120 sec] | *t*(18) = -1.21, uncorr. *p* = .243, FDR-corr. *p* = .543, BF = 0.38, *d* = 0.23 | *t*(22) = -1.88, uncorr. *p* = .073, FDR-corr. *p* = .252, BF = 0.94, *d* = 0.35 | ***t*(25) = -0.91, uncorr. *p* = .372, FDR-corr. *p* = .708, BF = 0.29, *d* = 0.17** | ***t*(24) = -3.76, uncorr. *p* = .001, FDR-corr. *p* = .005, BF = 40.84, *d* = 0.70** | ***t*(91) = -3.53, uncorr. *p* = .001, FDR-corr. *p* = .004, BF = 33.94, *d* = 0.66** | *t*(23) = -2.72, uncorr. *p* = .012, FDR-corr. *p* = .054, BF = 4.19, *d* = 0.51 | ***t*(23) = -0.70, uncorr. *p* = .490, FDR-corr. *p* = .783, BF = 0.25, *d* = 0.13** |
| RSA [130-180 sec] | *t*(17) = -1.60, uncorr. *p* = .129, FDR-corr. *p* = .358, BF = 0.61, *d* = 0.30 | ***t*(23) = -3.79, uncorr. *p* = .001, FDR-corr. *p* = .006, BF = 42.06, *d* = 0.71** | *t*(25) = -1.76, uncorr. *p* = .090, FDR-corr. *p* = .276, BF = 0.78, *d* = 0.33 | ***t*(25) = -3.52, uncorr. *p* = .002, FDR-corr. *p* = .009, BF = 23.45, *d* = 0.66** | ***t*(92) = -5.17, uncorr. *p* < .001, FDR-corr. *p* < .001, BF = 12979.06, *d* = 0.96** | *t*(22) = -2.43, uncorr. *p* = .024, FDR-corr. *p* = .096, BF = 2.38, *d* = 0.45 | *t*(21) = -1.96, uncorr. *p* = .063, FDR-corr. *p* = .226, BF = 1.07, *d* = 0.37 |
| RSA (corrected for RR) [0-60 sec] | *t*(15) = -1.40, uncorr. *p* = .181, FDR-corr. *p* = .451, BF = 0.47, *d* = 0.26 | *t*(18) = -1.82, uncorr. *p* = .085, FDR-corr. *p* = .272, BF = 0.86, *d* = 0.34 | ***t*(24) = -0.18, uncorr. *p* = .860, FDR-corr. *p* = .989, BF = 0.20, *d* = 0.03** | ***t*(24) = -3.33, uncorr. *p* = .003, FDR-corr. *p* = .014, BF = 15.23, *d* = 0.62** | ***t*(75) = -3.08, uncorr. *p* = .003, FDR-corr. *p* = .014, BF = 8.84, *d* = 0.57** | *t*(21) = -1.89, uncorr. *p* = .072, FDR-corr. *p* = .252, BF = 0.95, *d* = 0.35 | *t*(20) = -1.10, uncorr. *p* = .286, FDR-corr. *p* = .608, BF = 0.35, *d* = 0.20 |
| RSA (corrected for RR) [70-120 sec] | *t*(15) = -1.65, uncorr. *p* = .119, FDR-corr. *p* = .340, BF = 0.65, *d* = 0.31 | *t*(20) = -1.71, uncorr. *p* = .102, FDR-corr. *p* = .299, BF = 0.73, *d* = 0.32 | ***t*(24) = -0.51, uncorr. *p* = .612, FDR-corr. *p* = .836, BF = 0.23, *d* = 0.10** | ***t*(24) = -3.50, uncorr. *p* = .002, FDR-corr. *p* = .009, BF = 22.48, *d* = 0.65** | ***t*(88) = -3.38, uncorr. *p* = .001, FDR-corr. *p* = .006, BF = 21.26, *d* = 0.63** | *t*(20) = -1.75, uncorr. *p* = .095, FDR-corr. *p* = .282, BF = 0.77, *d* = 0.33 | ***t*(21) = -0.49, uncorr. *p* = .631, FDR-corr. *p* = .843, BF = 0.22, *d* = 0.09** |
| RSA (corrected for RR) [130-180 sec] | *t*(15) = -1.22, uncorr. *p* = .242, FDR-corr. *p* = .543, BF = 0.38, *d* = 0.23 | *t*(19) = -2.66, uncorr. *p* = .015, FDR-corr. *p* = .067, BF = 3.66, *d* = 0.50 | ***t*(24) = -0.79, uncorr. *p* = .437, FDR-corr. *p* = .744, BF = 0.27, *d* = 0.15** | *t*(24) = -2.35, uncorr. *p* = .027, FDR-corr. *p* = .105, BF = 2.04, *d* = 0.44 | ***t*(79) = -3.27, uncorr. *p* = .002, FDR-corr. *p* = .008, BF = 15.39, *d* = 0.61** | *t*(21) = -1.96, uncorr. *p* = .064, FDR-corr. *p* = .227, BF = 1.06, *d* = 0.37 | *t*(20) = -1.69, uncorr. *p* = .106, FDR-corr. *p* = .308, BF = 0.70, *d* = 0.32 |
| CO [0-60 sec] | ***t*(13) = 0.26, uncorr. *p* = .798, FDR-corr. *p* = .967, BF = 0.20, *d* = 0.05** | ***t*(14) = 0.60, uncorr. *p* = .557, FDR-corr. *p* = .831, BF = 0.24, *d* = 0.11** | ***t*(13) = 0.02, uncorr. *p* = .984, FDR-corr. *p* = .997, BF = 0.20, *d* < 0.01** | *t*(25) = 1.54, uncorr. *p* = .136, FDR-corr. *p* = .358, BF = 0.57, *d* = 0.29 | ***t*(54) = 0.82, uncorr. *p* = .414, FDR-corr. *p* = .719, BF = 0.14, *d* = 0.15** | ***t*(13) = 0.57, uncorr. *p* = .582, FDR-corr. *p* = .835, BF = 0.23, *d* = 0.11** | ***t*(14) = -0.49, uncorr. *p* = .633, FDR-corr. *p* = .843, BF = 0.22, *d* = 0.09** |
| CO [70-120 sec] | ***t*(14) = 0.38, uncorr. *p* = .708, FDR-corr. *p* = .900, BF = 0.21, *d* = 0.07** | ***t*(15) = 0.58, uncorr. *p* = .568, FDR-corr. *p* = .835, BF = 0.23, *d* = 0.11** | ***t*(15) = -0.61, uncorr. *p* = .550, FDR-corr. *p* = .831, BF = 0.24, *d* = 0.11** | ***t*(25) = 0.81, uncorr. *p* = .428, FDR-corr. *p* = .737, BF = 0.27, *d* = 0.15** | ***t*(64) = 0.54, uncorr. *p* = .592, FDR-corr. *p* = .835, BF = 0.12, *d* = 0.10** | ***t*(15) = 0.52, uncorr. *p* = .608, FDR-corr. *p* = .835, BF = 0.23, *d* = 0.10** | ***t*(15) = -0.02, uncorr. *p* = .985, FDR-corr. *p* = .997, BF = 0.20, *d* < 0.01** |
| CO [130-180 sec] | ***t*(13) = 0.66, uncorr. *p* = .524, FDR-corr. *p* = .808, BF = 0.24, *d* = 0.12** | ***t*(14) = 0.90, uncorr. *p* = .386, FDR-corr. *p* = .710, BF = 0.29, *d* = 0.17** | ***t*(15) = -0.18, uncorr. *p* = .861, FDR-corr. *p* = .989, BF = 0.20, *d* = 0.03** | *t*(24) = 1.29, uncorr. *p* = .210, FDR-corr. *p* = .501, BF = 0.42, *d* = 0.24 | ***t*(57) = 1.21, uncorr. *p* = .231, FDR-corr. *p* = .533, BF = 0.21, *d* = 0.23** | ***t*(14) = 0.56, uncorr. *p* = .586, FDR-corr. *p* = .835, BF = 0.23, *d* = 0.10** | ***t*(14) = 0.08, uncorr. *p* = .936, FDR-corr. *p* = .997, BF = 0.20, *d* = 0.02** |
| TPR [0-60 sec] | ***t*(14) = -0.03, uncorr. *p* = .974, FDR-corr. *p* = .997, BF = 0.19, *d* = 0.01** | ***t*(18) = -0.88, uncorr. *p* = .393, FDR-corr. *p* = .710, BF = 0.28, *d* = 0.16** | ***t*(16) = 0.61, uncorr. *p* = .552, FDR-corr. *p* = .831, BF = 0.24, *d* = 0.11** | ***t*(24) = 0.41, uncorr. *p* = .682, FDR-corr. *p* = .880, BF = 0.21, *d* = 0.08** | ***t*(63) < 0.01, uncorr. *p* = .996, FDR-corr. *p* = .997, BF = 0.10, *d* < 0.01** | ***t*(16) = -0.70, uncorr. *p* = .491, FDR-corr. *p* = .783, BF = 0.25, *d* = 0.13** | ***t*(21) = 0.35, uncorr. *p* = .727, FDR-corr. *p* = .912, BF = 0.21, *d* = 0.07** |
| TPR [70-120 sec] | ***t*(15) = 0.16, uncorr. *p* = .875, FDR-corr. *p* = .992, BF = 0.20, *d* = 0.03** | ***t*(18) = -0.75, uncorr. *p* = .462, FDR-corr. *p* = .768, BF = 0.26, *d* = 0.14** | ***t*(14) = 0.85, uncorr. *p* = .412, FDR-corr. *p* = .719, BF = 0.28, *d* = 0.16** | ***t*(23) = -0.03, uncorr. *p* = .974, FDR-corr. *p* = .997, BF = 0.20, *d* = 0.01** | ***t*(63) = 0.15, uncorr. *p* = .883, FDR-corr. *p* = .992, BF = 0.10, *d* = 0.03** | ***t*(18) = -0.75, uncorr. *p* = .464, FDR-corr. *p* = .768, BF = 0.26, *d* = 0.14** | ***t*(20) = -0.17, uncorr. *p* = .866, FDR-corr. *p* = .989, BF = 0.20, *d* = 0.03** |
| TPR [130-180 sec] | ***t*(15) = -0.07, uncorr. *p* = .943, FDR-corr. *p* = .997, BF = 0.19, *d* = 0.01** | *t*(17) = -0.99, uncorr. *p* = .336, FDR-corr. *p* = .670, BF = 0.31, *d* = 0.18 | ***t*(15) = 0.23, uncorr. *p* = .821, FDR-corr. *p* = .979, BF = 0.21, *d* = 0.04** | *t*(22) = -1.84, uncorr. *p* = .080, FDR-corr. *p* = .261, BF = 0.86, *d* = 0.34 | ***t*(66) = -0.91, uncorr. *p* = .368, FDR-corr. *p* = .708, BF = 0.15, *d* = 0.17** | ***t*(18) = -0.60, uncorr. *p* = .559, FDR-corr. *p* = .831, BF = 0.24, *d* = 0.11** | ***t*(19) < 0.01, uncorr. *p* = .996, FDR-corr. *p* = .997, BF = 0.20, *d* < 0.01** |

*Note*. Results with significant *p*-values after FDR-correction are displayed in bold black font, and results with substantial Bayesian evidence for the null hypothesis are displayed in bold orange font, with inconclusive results in regular font. FDR = false-discovery rate; BF = Bayes Factor; *d* = Cohen’s *d*; BP = blood pressure; HR = heart rate; SC = skin conductance; PEP = pre-ejection period; RR = respiratory rate; RSA = respiratory sinus arrhythmia; CO = cardiac output; TPR = total peripheral resistance.

Table S2. Results of two sample *t*-tests of reactivity comparing separate conditions against the control condition (TV salience).

|  | Mortality salience | Freedom restriction | Uncontroll-ability | Uncertainty | Existential threat composite | Social-evaluative threat |
| --- | --- | --- | --- | --- | --- | --- |
| Positive affect | *t*(158) = -1.66, uncorr. *p* = .099, FDR-corr. *p* = .963, BF = 0.92, *d* = 0.44 | ***t*(158) = -0.40, uncorr. *p* = .688, FDR-corr. *p* = .974, BF = 0.30, *d* = 0.11** | *t*(158) = -2.65, uncorr. *p* = .009, FDR-corr. *p* = .204, BF = 7.88, *d* = 0.71 | *t*(158) = -2.06, uncorr. *p* = .041, FDR-corr. *p* = .541, BF = 2.57, *d* = 0.55 | *t*(158) = -2.10, uncorr. *p* = .037, FDR-corr. *p* = .528, BF = 2.19, *d* = 0.44 | *t*(158) = 3.04, uncorr. *p* = .003, FDR-corr. *p* = .102, BF = 8.64, *d* = 0.81 |
| Negative affect | *t*(161) = 1.63, uncorr. *p* = .105, FDR-corr. *p* = .963, BF = 1.64, *d* = 0.43 | ***t*(161) = 4.25, uncorr. *p* < .001, FDR-corr. *p* = .002, BF = 664.42, *d* = 1.14** | ***t*(161) = 4.86, uncorr. *p* < .001, FDR-corr. *p* = .001, BF = 413.12, *d* = 1.30** | ***t*(161) = 4.13, uncorr. *p* < .001, FDR-corr. *p* = .003, BF = 26862.14, *d* = 1.09** | ***t*(163) = 4.51, uncorr. *p* < .001, FDR-corr. *p* = .001, BF = 1381.60, *d* = 0.95** | *t*(161) = 1.23, uncorr. *p* = .222, FDR-corr. *p* = .974, BF = 0.65, *d* = 0.33 |
| Positive affect-related words | W = 382, uncorr. *p* = .491, FDR-corr. *p* = .702, *d* = 0.18 | W = 344, uncorr. *p* = .351, FDR-corr. *p* = .527, *d* = 0.25 | W = 366, uncorr. *p* = .614, FDR-corr. *p* = .768, *d* = 0.13 | W = 324, uncorr. *p* = .135, FDR-corr. *p* = .353, *d* = 0.41 | W = 1416, uncorr. *p* = .258, FDR-corr. *p* = .456, *d* = 0.24 | W = 279, uncorr. *p* = .041, FDR-corr. *p* = .139, *d* = 0.60 |
| Negative affect-related words | **W = 120, uncorr. *p* < .001, FDR-corr. *p* < .001, *d* = 2.03** | W = 368, uncorr. *p* = .605, FDR-corr. *p* = .768, *d* = 0.13 | W = 282, uncorr. *p* = .034, FDR-corr. *p* = .139, *d* = 0.58 | W = 312, uncorr. *p* = .072, FDR-corr. *p* = .215, *d* = 0.47 | **W = 1083, uncorr. *p* = .003, FDR-corr. *p* = .034, *d* = 0.69** | W = 382, uncorr. *p* = .833, FDR-corr. *p* = 1.000, *d* = 0.05 |
| Anger-related words | W = 420, uncorr. *p* = NaN, FDR-corr. *p* = NaN, *d* < 0.01 | W = 364, uncorr. *p* = .161, FDR-corr. *p* = .353, *d* = 0.14 | W = 336, uncorr. *p* = .042, FDR-corr. *p* = .139, *d* = 0.29 | W = 392, uncorr. *p* = .343, FDR-corr. *p* = .527, *d* = 0.07 | W = 1512, uncorr. *p* = .185, FDR-corr. *p* = .369, *d* = 0.12 | W = 392, uncorr. *p* = NaN, FDR-corr. *p* = NaN, *d* < 0.01 |
| Fear/anxiety-related words | W = 366, uncorr. *p* = .165, FDR-corr. *p* = .353, *d* = 0.26 | W = 407, uncorr. *p* = .543, FDR-corr. *p* = .740, *d* = 0.08 | W = 393, uncorr. *p* = .985, FDR-corr. *p* = 1.000, *d* = 0.01 | W = 307, uncorr. *p* = .022, FDR-corr. *p* = .112, *d* = 0.50 | W = 1473, uncorr. *p* = .250, FDR-corr. *p* = .456, *d* = 0.17 | W = 392, uncorr. *p* = 1.000, FDR-corr. *p* = 1.000, *d* < 0.01 |
| Sadness-related words | **W = 198, uncorr. *p* < .001, FDR-corr. *p* < .001, *d* = 1.25** | W = 362, uncorr. *p* = .287, FDR-corr. *p* = .479, *d* = 0.15 | W = 294, uncorr. *p* = .012, FDR-corr. *p* = .075, *d* = 0.52 | W = 362, uncorr. *p* = .162, FDR-corr. *p* = .353, *d* = 0.22 | W = 1216, uncorr. *p* = .007, FDR-corr. *p* = .051, *d* = 0.50 | W = 392, uncorr. *p* = 1.000, FDR-corr. *p* = 1.000, *d* < 0.01 |
| Subjective arousal [0-60 sec] | *t*(149) = 0.62, uncorr. *p* = .538, FDR-corr. *p* = .974, BF = 0.32, *d* = 0.16 | *t*(152) = 1.75, uncorr. *p* = .081, FDR-corr. *p* = .889, BF = 1.39, *d* = 0.47 | *t*(155) = 0.81, uncorr. *p* = .419, FDR-corr. *p* = .974, BF = 0.41, *d* = 0.22 | *t*(152) = 0.85, uncorr. *p* = .396, FDR-corr. *p* = .974, BF = 0.38, *d* = 0.23 | *t*(153) = 1.26, uncorr. *p* = .210, FDR-corr. *p* = .974, BF = 0.51, *d* = 0.27 | *t*(154) = 2.85, uncorr. *p* = .005, FDR-corr. *p* = .134, BF = 11.20, *d* = 0.76 |
| Subjective arousal [70-120 sec] | *t*(151) = 1.14, uncorr. *p* = .258, FDR-corr. *p* = .974, BF = 0.48, *d* = 0.30 | *t*(150) = 2.13, uncorr. *p* = .035, FDR-corr. *p* = .528, BF = 3.70, *d* = 0.57 | *t*(154) = 1.15, uncorr. *p* = .251, FDR-corr. *p* = .974, BF = 0.54, *d* = 0.31 | *t*(152) = 0.73, uncorr. *p* = .465, FDR-corr. *p* = .974, BF = 0.36, *d* = 0.19 | *t*(153) = 1.61, uncorr. *p* = .109, FDR-corr. *p* = .963, BF = 0.80, *d* = 0.34 | *t*(156) = 2.20, uncorr. *p* = .029, FDR-corr. *p* = .489, BF = 4.21, *d* = 0.59 |
| Subjective arousal [130-180 sec] | *t*(154) = 0.83, uncorr. *p* = .406, FDR-corr. *p* = .974, BF = 0.42, *d* = 0.22 | *t*(152) = 2.88, uncorr. *p* = .004, FDR-corr. *p* = .134, BF = 34.98, *d* = 0.77 | *t*(156) = 1.96, uncorr. *p* = .051, FDR-corr. *p* = .597, BF = 1.73, *d* = 0.52 | *t*(148) = 1.49, uncorr. *p* = .138, FDR-corr. *p* = .974, BF = 1.22, *d* = 0.39 | *t*(155) = 2.22, uncorr. *p* = .028, FDR-corr. *p* = .489, BF = 2.76, *d* = 0.47 | *t*(156) = 2.22, uncorr. *p* = .028, FDR-corr. *p* = .489, BF = 3.80, *d* = 0.59 |
| BP [pre] | *t*(112) = 0.37, uncorr. *p* = .710, FDR-corr. *p* = .974, BF = 0.32, *d* = 0.10 | ***t*(114) = -0.11, uncorr. *p* = .912, FDR-corr. *p* = .974, BF = 0.29, *d* = 0.03** | *t*(114) = 0.68, uncorr. *p* = .500, FDR-corr. *p* = .974, BF = 0.38, *d* = 0.18 | *t*(113) = 1.16, uncorr. *p* = .250, FDR-corr. *p* = .974, BF = 0.52, *d* = 0.31 | *t*(94) = 0.62, uncorr. *p* = .535, FDR-corr. *p* = .974, BF = 0.43, *d* = 0.13 | ***t*(114) = 0.11, uncorr. *p* = .916, FDR-corr. *p* = .974, BF = 0.30, *d* = 0.03** |
| BP [post] | ***t*(128) = -0.29, uncorr. *p* = .771, FDR-corr. *p* = .974, BF = 0.29, *d* = 0.08** | ***t*(129) = -0.39, uncorr. *p* = .694, FDR-corr. *p* = .974, BF = 0.30, *d* = 0.11** | ***t*(129) = -0.30, uncorr. *p* = .768, FDR-corr. *p* = .974, BF = 0.30, *d* = 0.08** | *t*(128) = -0.88, uncorr. *p* = .383, FDR-corr. *p* = .974, BF = 0.38, *d* = 0.23 | *t*(113) = -0.56, uncorr. *p* = .575, FDR-corr. *p* = .974, BF = 0.34, *d* = 0.12 | *t*(129) = -0.77, uncorr. *p* = .445, FDR-corr. *p* = .974, BF = 0.33, *d* = 0.20 |
| HR [0-60 sec] | *t*(48) = 0.51, uncorr. *p* = .613, FDR-corr. *p* = .974, BF = 0.68, *d* = 0.13 | *t*(90) = 1.05, uncorr. *p* = .296, FDR-corr. *p* = .974, BF = 1.47, *d* = 0.28 | *t*(89) = 0.45, uncorr. *p* = .656, FDR-corr. *p* = .974, BF = 0.49, *d* = 0.12 | *t*(114) = 1.34, uncorr. *p* = .184, FDR-corr. *p* = .974, BF = 1.87, *d* = 0.35 | *t*(77) = 1.00, uncorr. *p* = .319, FDR-corr. *p* = .974, BF = 1.36, *d* = 0.21 | *t*(84) = 0.89, uncorr. *p* = .378, FDR-corr. *p* = .974, BF = 1.11, *d* = 0.24 |
| HR [70-120 sec] | *t*(60) = 0.26, uncorr. *p* = .799, FDR-corr. *p* = .974, BF = 0.40, *d* = 0.07 | *t*(98) = 0.60, uncorr. *p* = .550, FDR-corr. *p* = .974, BF = 0.49, *d* = 0.16 | *t*(91) = -0.30, uncorr. *p* = .763, FDR-corr. *p* = .974, BF = 0.38, *d* = 0.08 | *t*(119) = 0.52, uncorr. *p* = .606, FDR-corr. *p* = .974, BF = 0.39, *d* = 0.14 | *t*(88) = 0.33, uncorr. *p* = .743, FDR-corr. *p* = .974, BF = 0.32, *d* = 0.07 | *t*(89) = 0.60, uncorr. *p* = .551, FDR-corr. *p* = .974, BF = 0.46, *d* = 0.16 |
| HR [130-180 sec] | *t*(60) = 0.44, uncorr. *p* = .660, FDR-corr. *p* = .974, BF = 0.51, *d* = 0.12 | *t*(93) = 0.63, uncorr. *p* = .533, FDR-corr. *p* = .974, BF = 0.52, *d* = 0.17 | *t*(90) = -0.05, uncorr. *p* = .958, FDR-corr. *p* = .974, BF = 0.34, *d* = 0.01 | *t*(123) = 0.84, uncorr. *p* = .404, FDR-corr. *p* = .974, BF = 0.52, *d* = 0.22 | *t*(85) = 0.57, uncorr. *p* = .572, FDR-corr. *p* = .974, BF = 0.42, *d* = 0.12 | *t*(96) = 0.61, uncorr. *p* = .546, FDR-corr. *p* = .974, BF = 0.48, *d* = 0.16 |
| SC [0-60 sec] | *t*(90) = 1.46, uncorr. *p* = .149, FDR-corr. *p* = .974, BF = 1.07, *d* = 0.38 | *t*(73) = 1.49, uncorr. *p* = .142, FDR-corr. *p* = .974, BF = 3.51, *d* = 0.40 | *t*(84) = 0.73, uncorr. *p* = .468, FDR-corr. *p* = .974, BF = 0.56, *d* = 0.19 | *t*(95) = -0.18, uncorr. *p* = .858, FDR-corr. *p* = .974, BF = 0.32, *d* = 0.05 | *t*(89) = 1.12, uncorr. *p* = .266, FDR-corr. *p* = .974, BF = 1.29, *d* = 0.24 | *t*(90) = 0.11, uncorr. *p* = .912, FDR-corr. *p* = .974, BF = 0.32, *d* = 0.03 |
| SC [70-120 sec] | *t*(73) = 1.52, uncorr. *p* = .134, FDR-corr. *p* = .974, BF = 1.96, *d* = 0.40 | *t*(83) = 2.00, uncorr. *p* = .049, FDR-corr. *p* = .597, BF = 15.85, *d* = 0.53 | *t*(78) = 1.20, uncorr. *p* = .233, FDR-corr. *p* = .974, BF = 2.06, *d* = 0.32 | *t*(93) = 0.23, uncorr. *p* = .818, FDR-corr. *p* = .974, BF = 0.40, *d* = 0.06 | *t*(82) = 1.55, uncorr. *p* = .126, FDR-corr. *p* = .974, BF = 3.59, *d* = 0.33 | *t*(90) = 1.19, uncorr. *p* = .239, FDR-corr. *p* = .974, BF = 1.28, *d* = 0.32 |
| SC [130-180 sec] | *t*(75) = 0.42, uncorr. *p* = .674, FDR-corr. *p* = .974, BF = 0.42, *d* = 0.11 | *t*(74) = 0.88, uncorr. *p* = .384, FDR-corr. *p* = .974, BF = 0.67, *d* = 0.23 | *t*(75) = 0.34, uncorr. *p* = .735, FDR-corr. *p* = .974, BF = 0.42, *d* = 0.09 | *t*(89) = -0.22, uncorr. *p* = .826, FDR-corr. *p* = .974, BF = 0.35, *d* = 0.06 | *t*(85) = 0.47, uncorr. *p* = .643, FDR-corr. *p* = .974, BF = 0.36, *d* = 0.10 | *t*(99) = 0.22, uncorr. *p* = .829, FDR-corr. *p* = .974, BF = 0.32, *d* = 0.06 |
| PEP [0-60 sec] | *t*(36) = -0.23, uncorr. *p* = .822, FDR-corr. *p* = .974, BF = 0.69, *d* = 0.06 | *t*(71) = -0.05, uncorr. *p* = .958, FDR-corr. *p* = .974, BF = 0.42, *d* = 0.01 | *t*(67) = -0.53, uncorr. *p* = .599, FDR-corr. *p* = .974, BF = 1.31, *d* = 0.14 | *t*(108) = -0.43, uncorr. *p* = .670, FDR-corr. *p* = .974, BF = 0.55, *d* = 0.11 | *t*(60) = -0.37, uncorr. *p* = .711, FDR-corr. *p* = .974, BF = 0.47, *d* = 0.08 | *t*(72) = -0.06, uncorr. *p* = .949, FDR-corr. *p* = .974, BF = 0.44, *d* = 0.02 |
| PEP [70-120 sec] | *t*(40) = -0.21, uncorr. *p* = .834, FDR-corr. *p* = .974, BF = 0.76, *d* = 0.06 | *t*(72) = -0.15, uncorr. *p* = .882, FDR-corr. *p* = .974, BF = 0.44, *d* = 0.04 | *t*(67) = -0.46, uncorr. *p* = .644, FDR-corr. *p* = .974, BF = 1.07, *d* = 0.12 | *t*(107) = -0.25, uncorr. *p* = .804, FDR-corr. *p* = .974, BF = 0.43, *d* = 0.07 | *t*(63) = -0.33, uncorr. *p* = .741, FDR-corr. *p* = .974, BF = 0.45, *d* = 0.07 | *t*(70) = -0.08, uncorr. *p* = .938, FDR-corr. *p* = .974, BF = 0.43, *d* = 0.02 |
| PEP [130-180 sec] | *t*(39) = -0.48, uncorr. *p* = .636, FDR-corr. *p* = .974, BF = 1.25, *d* = 0.13 | *t*(73) = 0.14, uncorr. *p* = .891, FDR-corr. *p* = .974, BF = 0.44, *d* = 0.04 | *t*(71) = -0.35, uncorr. *p* = .729, FDR-corr. *p* = .974, BF = 0.58, *d* = 0.09 | *t*(109) = -0.09, uncorr. *p* = .926, FDR-corr. *p* = .974, BF = 0.35, *d* = 0.02 | *t*(64) = -0.28, uncorr. *p* = .780, FDR-corr. *p* = .974, BF = 0.37, *d* = 0.06 | *t*(69) = 0.02, uncorr. *p* = .986, FDR-corr. *p* = .986, BF = 0.40, *d* < 0.01 |
| RR [0-60 sec] | *t*(122) = -0.47, uncorr. *p* = .640, FDR-corr. *p* = .974, BF = 0.38, *d* = 0.12 | *t*(147) = 0.59, uncorr. *p* = .554, FDR-corr. *p* = .974, BF = 0.34, *d* = 0.16 | *t*(152) = 0.64, uncorr. *p* = .521, FDR-corr. *p* = .974, BF = 0.35, *d* = 0.17 | *t*(147) = 1.17, uncorr. *p* = .243, FDR-corr. *p* = .974, BF = 0.59, *d* = 0.31 | ***t*(142) = 0.58, uncorr. *p* = .565, FDR-corr. *p* = .974, BF = 0.28, *d* = 0.12** | ***t*(149) = -0.41, uncorr. *p* = .679, FDR-corr. *p* = .974, BF = 0.31, *d* = 0.11** |
| RR [70-120 sec] | *t*(115) = -1.04, uncorr. *p* = .300, FDR-corr. *p* = .974, BF = 0.72, *d* = 0.27 | ***t*(145) = 0.16, uncorr. *p* = .876, FDR-corr. *p* = .974, BF = 0.28, *d* = 0.04** | ***t*(150) = 0.31, uncorr. *p* = .754, FDR-corr. *p* = .974, BF = 0.29, *d* = 0.08** | *t*(144) = 0.64, uncorr. *p* = .521, FDR-corr. *p* = .974, BF = 0.34, *d* = 0.17 | ***t*(137) = -0.02, uncorr. *p* = .986, FDR-corr. *p* = .986, BF = 0.23, *d* < 0.01** | *t*(145) = 1.09, uncorr. *p* = .279, FDR-corr. *p* = .974, BF = 0.70, *d* = 0.29 |
| RR [130-180 sec] | ***t*(121) = 0.16, uncorr. *p* = .875, FDR-corr. *p* = .974, BF = 0.31, *d* = 0.04** | *t*(143) = 1.06, uncorr. *p* = .293, FDR-corr. *p* = .974, BF = 0.49, *d* = 0.28 | *t*(153) = 0.97, uncorr. *p* = .333, FDR-corr. *p* = .974, BF = 0.42, *d* = 0.26 | *t*(148) = 1.68, uncorr. *p* = .096, FDR-corr. *p* = .963, BF = 1.16, *d* = 0.44 | *t*(141) = 1.18, uncorr. *p* = .238, FDR-corr. *p* = .974, BF = 0.48, *d* = 0.25 | ***t*(148) = 0.15, uncorr. *p* = .877, FDR-corr. *p* = .974, BF = 0.29, *d* = 0.04** |
| RSA [0-60 sec] | ***t*(100) = 0.07, uncorr. *p* = .942, FDR-corr. *p* = .974, BF = 0.31, *d* = 0.02** | *t*(127) = -0.61, uncorr. *p* = .542, FDR-corr. *p* = .974, BF = 0.35, *d* = 0.16 | *t*(147) = 0.57, uncorr. *p* = .567, FDR-corr. *p* = .974, BF = 0.33, *d* = 0.15 | *t*(143) = -1.22, uncorr. *p* = .226, FDR-corr. *p* = .974, BF = 0.61, *d* = 0.32 | ***t*(129) = -0.36, uncorr. *p* = .718, FDR-corr. *p* = .974, BF = 0.26, *d* = 0.08** | ***t*(130) = -0.27, uncorr. *p* = .786, FDR-corr. *p* = .974, BF = 0.30, *d* = 0.07** |
| RSA [70-120 sec] | *t*(107) = -0.33, uncorr. *p* = .744, FDR-corr. *p* = .974, BF = 0.32, *d* = 0.09 | *t*(134) = -0.71, uncorr. *p* = .479, FDR-corr. *p* = .974, BF = 0.35, *d* = 0.19 | ***t*(149) = 0.03, uncorr. *p* = .979, FDR-corr. *p* = .986, BF = 0.28, *d* = 0.01** | *t*(145) = -1.24, uncorr. *p* = .217, FDR-corr. *p* = .974, BF = 0.59, *d* = 0.33 | *t*(133) = -0.70, uncorr. *p* = .483, FDR-corr. *p* = .974, BF = 0.32, *d* = 0.15 | *t*(135) = -1.45, uncorr. *p* = .150, FDR-corr. *p* = .974, BF = 0.65, *d* = 0.39 |
| RSA [130-180 sec] | *t*(96) = 0.09, uncorr. *p* = .925, FDR-corr. *p* = .974, BF = 0.32, *d* = 0.02 | *t*(131) = -1.29, uncorr. *p* = .200, FDR-corr. *p* = .974, BF = 0.69, *d* = 0.34 | ***t*(147) = 0.19, uncorr. *p* = .851, FDR-corr. *p* = .974, BF = 0.28, *d* = 0.05** | *t*(142) = -0.84, uncorr. *p* = .400, FDR-corr. *p* = .974, BF = 0.41, *d* = 0.22 | ***t*(127) = -0.56, uncorr. *p* = .578, FDR-corr. *p* = .974, BF = 0.29, *d* = 0.12** | ***t*(132) = -0.11, uncorr. *p* = .913, FDR-corr. *p* = .974, BF = 0.29, *d* = 0.03** |
| RSA (corrected for RR) [0-60 sec] | ***t*(84) = -0.13, uncorr. *p* = .896, FDR-corr. *p* = .974, BF = 0.31, *d* = 0.03** | *t*(114) = -0.39, uncorr. *p* = .696, FDR-corr. *p* = .974, BF = 0.35, *d* = 0.10 | *t*(140) = 0.94, uncorr. *p* = .347, FDR-corr. *p* = .974, BF = 0.33, *d* = 0.25 | *t*(133) = -0.76, uncorr. *p* = .451, FDR-corr. *p* = .974, BF = 0.61, *d* = 0.20 | ***t*(116) = -0.12, uncorr. *p* = .903, FDR-corr. *p* = .974, BF = 0.26, *d* = 0.03** | ***t*(123) = -0.49, uncorr. *p* = .623, FDR-corr. *p* = .974, BF = 0.30, *d* = 0.13** |
| RSA (corrected for RR) [70-120 sec] | *t*(97) = -0.70, uncorr. *p* = .484, FDR-corr. *p* = .974, BF = 0.32, *d* = 0.18 | *t*(127) = -0.69, uncorr. *p* = .492, FDR-corr. *p* = .974, BF = 0.35, *d* = 0.18 | ***t*(146) = 0.14, uncorr. *p* = .885, FDR-corr. *p* = .974, BF = 0.28, *d* = 0.04** | *t*(142) = -1.07, uncorr. *p* = .286, FDR-corr. *p* = .974, BF = 0.59, *d* = 0.28 | *t*(127) = -0.75, uncorr. *p* = .457, FDR-corr. *p* = .974, BF = 0.32, *d* = 0.16 | *t*(132) = -1.14, uncorr. *p* = .258, FDR-corr. *p* = .974, BF = 0.65, *d* = 0.30 |
| RSA (corrected for RR) [130-180 sec] | *t*(88) = 0.15, uncorr. *p* = .882, FDR-corr. *p* = .974, BF = 0.32, *d* = 0.04 | *t*(126) = -0.98, uncorr. *p* = .328, FDR-corr. *p* = .974, BF = 0.69, *d* = 0.26 | ***t*(143) = 0.56, uncorr. *p* = .578, FDR-corr. *p* = .974, BF = 0.28, *d* = 0.15** | *t*(135) = -0.27, uncorr. *p* = .789, FDR-corr. *p* = .974, BF = 0.41, *d* = 0.07 | ***t*(122) = -0.16, uncorr. *p* = .875, FDR-corr. *p* = .974, BF = 0.29, *d* = 0.03** | ***t*(125) = -0.07, uncorr. *p* = .948, FDR-corr. *p* = .974, BF = 0.29, *d* = 0.02** |
| CO [0-60 sec] | *t*(42) = 0.50, uncorr. *p* = .623, FDR-corr. *p* = .974, BF = 0.78, *d* = 0.13 | *t*(73) = 0.71, uncorr. *p* = .479, FDR-corr. *p* = .974, BF = 1.04, *d* = 0.19 | *t*(84) = 0.35, uncorr. *p* = .724, FDR-corr. *p* = .974, BF = 0.45, *d* = 0.09 | *t*(105) = 0.95, uncorr. *p* = .345, FDR-corr. *p* = .974, BF = 1.29, *d* = 0.25 | *t*(69) = 0.77, uncorr. *p* = .444, FDR-corr. *p* = .974, BF = 0.73, *d* = 0.16 | *t*(77) = 0.69, uncorr. *p* = .491, FDR-corr. *p* = .974, BF = 0.92, *d* = 0.18 |
| CO [70-120 sec] | *t*(52) = 0.35, uncorr. *p* = .731, FDR-corr. *p* = .974, BF = 0.52, *d* = 0.09 | *t*(79) = 0.39, uncorr. *p* = .697, FDR-corr. *p* = .974, BF = 0.45, *d* = 0.10 | *t*(93) = -0.37, uncorr. *p* = .709, FDR-corr. *p* = .974, BF = 0.42, *d* = 0.10 | *t*(112) = 0.38, uncorr. *p* = .704, FDR-corr. *p* = .974, BF = 0.37, *d* = 0.10 | *t*(78) = 0.25, uncorr. *p* = .804, FDR-corr. *p* = .974, BF = 0.32, *d* = 0.05 | *t*(79) = 0.35, uncorr. *p* = .725, FDR-corr. *p* = .974, BF = 0.42, *d* = 0.09 |
| CO [130-180 sec] | *t*(43) = 0.52, uncorr. *p* = .608, FDR-corr. *p* = .974, BF = 0.67, *d* = 0.14 | *t*(83) = 0.54, uncorr. *p* = .590, FDR-corr. *p* = .974, BF = 0.57, *d* = 0.14 | *t*(94) = -0.17, uncorr. *p* = .862, FDR-corr. *p* = .974, BF = 0.37, *d* = 0.05 | *t*(110) = 0.57, uncorr. *p* = .570, FDR-corr. *p* = .974, BF = 0.44, *d* = 0.15 | *t*(74) = 0.48, uncorr. *p* = .636, FDR-corr. *p* = .974, BF = 0.38, *d* = 0.10 | *t*(75) = 0.31, uncorr. *p* = .756, FDR-corr. *p* = .974, BF = 0.41, *d* = 0.08 |
| TPR [0-60 sec] | *t*(61) = -0.28, uncorr. *p* = .779, FDR-corr. *p* = .974, BF = 0.38, *d* = 0.07 | *t*(99) = -0.84, uncorr. *p* = .401, FDR-corr. *p* = .974, BF = 0.46, *d* = 0.23 | ***t*(97) = 0.10, uncorr. *p* = .922, FDR-corr. *p* = .974, BF = 0.31, *d* = 0.03** | ***t*(134) = -0.14, uncorr. *p* = .892, FDR-corr. *p* = .974, BF = 0.29, *d* = 0.04** | ***t*(94) = -0.37, uncorr. *p* = .713, FDR-corr. *p* = .974, BF = 0.31, *d* = 0.08** | *t*(105) = -0.73, uncorr. *p* = .470, FDR-corr. *p* = .974, BF = 0.43, *d* = 0.19 |
| TPR [70-120 sec] | *t*(58) = 0.25, uncorr. *p* = .800, FDR-corr. *p* = .974, BF = 0.38, *d* = 0.07 | *t*(111) = -0.31, uncorr. *p* = .755, FDR-corr. *p* = .974, BF = 0.32, *d* = 0.08 | *t*(105) = 0.65, uncorr. *p* = .520, FDR-corr. *p* = .974, BF = 0.42, *d* = 0.17 | ***t*(129) = 0.14, uncorr. *p* = .887, FDR-corr. *p* = .974, BF = 0.29, *d* = 0.04** | ***t*(94) = 0.24, uncorr. *p* = .811, FDR-corr. *p* = .974, BF = 0.29, *d* = 0.05** | *t*(106) = -0.36, uncorr. *p* = .719, FDR-corr. *p* = .974, BF = 0.33, *d* = 0.10 |
| TPR [130-180 sec] | *t*(58) = -0.06, uncorr. *p* = .955, FDR-corr. *p* = .974, BF = 0.36, *d* = 0.01 | *t*(94) = -0.56, uncorr. *p* = .574, FDR-corr. *p* = .974, BF = 0.39, *d* = 0.15 | ***t*(103) = 0.14, uncorr. *p* = .886, FDR-corr. *p* = .974, BF = 0.31, *d* = 0.04** | *t*(122) = -0.75, uncorr. *p* = .457, FDR-corr. *p* = .974, BF = 0.43, *d* = 0.20 | *t*(87) = -0.36, uncorr. *p* = .721, FDR-corr. *p* = .974, BF = 0.34, *d* = 0.08 | *t*(101) = -0.42, uncorr. *p* = .678, FDR-corr. *p* = .974, BF = 0.34, *d* = 0.11 |

*Note*. Based on linear models. Results with significant *p*-values after FDR-correction are displayed in bold black font, and results with substantial Bayesian evidence for the null hypothesis are displayed in bold orange font, with inconclusive results in regular font. FDR = false-discovery rate; BF = Bayes Factor; *d* = Cohen’s *d*; BP = blood pressure; HR = heart rate; SC = skin conductance; PEP = pre-ejection period; RR = respiratory rate; RSA = respiratory sinus arrhythmia; CO = cardiac output; TPR = total peripheral resistance.

Table S3. Results of two sample *t*-tests of reactivity comparing existential threat conditions against the social-evaluative threat condition.

|  | Mortality salience | Freedom restriction | Uncontroll-ability | Uncertainty | Existential threat composite |
| --- | --- | --- | --- | --- | --- |
| Positive affect | ***t*(163) = -4.82, uncorr. *p* < .001, FDR-corr. *p* < .001, BF = 327.59, *d* = 1.27** | ***t*(163) = -3.49, uncorr. *p* = .001, FDR-corr. *p* = .016, BF = 17.72, *d* = 0.93** | ***t*(163) = -5.78, uncorr. *p* < .001, FDR-corr. *p* < .001, BF = 8171.41, *d* = 1.54** | ***t*(163) = -5.21, uncorr. *p* < .001, FDR-corr. *p* < .001, BF = 2528.10, *d* = 1.38** | ***t*(166) = -6.04, uncorr. *p* < .001, FDR-corr. *p* < .001, BF = 467495.10, *d* = 1.27** |
| Negative affect | ***t*(163) = 0.39, uncorr. *p* = .701, FDR-corr. *p* = .991, BF = 0.29, *d* = 0.10** | *t*(163) = 3.04, uncorr. *p* = .003, FDR-corr. *p* = .056, BF = 7.41, *d* = 0.81 | ***t*(163) = 3.65, uncorr. *p* < .001, FDR-corr. *p* = .011, BF = 10.08, *d* = 0.98** | *t*(163) = 2.91, uncorr. *p* = .004, FDR-corr. *p* = .070, BF = 20.78, *d* = 0.77 | *t*(166) = 3.03, uncorr. *p* = .003, FDR-corr. *p* = .056, BF = 8.18, *d* = 0.64 |
| Positive affect-related words | W = 515, uncorr. *p* = .113, FDR-corr. *p* = .268, *d* = 0.46 | W = 452, uncorr. *p* = .293, FDR-corr. *p* = .407, *d* = 0.31 | W = 476, uncorr. *p* = .137, FDR-corr. *p* = .268, *d* = 0.44 | W = 430, uncorr. *p* = .683, FDR-corr. *p* = .776, *d* = 0.12 | W = 1874, uncorr. *p* = .139, FDR-corr. *p* = .268, *d* = 0.33 |
| Negative affect-related words | **W = 124, uncorr. *p* < .001, FDR-corr. *p* < .001, *d* = 1.98** | W = 378, uncorr. *p* = .785, FDR-corr. *p* = .853, *d* = 0.07 | W = 288, uncorr. *p* = .049, FDR-corr. *p* = .153, *d* = 0.55 | W = 324, uncorr. *p* = .121, FDR-corr. *p* = .268, *d* = 0.41 | **W = 1116, uncorr. *p* = .006, FDR-corr. *p* = .046, *d* = 0.65** |
| Anger-related words | W = 420, uncorr. *p* = NaN, FDR-corr. *p* = NaN, *d* < 0.01 | W = 364, uncorr. *p* = .161, FDR-corr. *p* = .270, *d* = 0.14 | W = 336, uncorr. *p* = .042, FDR-corr. *p* = .149, *d* = 0.29 | W = 392, uncorr. *p* = .343, FDR-corr. *p* = .429, *d* = 0.07 | W = 1512, uncorr. *p* = .185, FDR-corr. *p* = .288, *d* = 0.12 |
| Fear/anxiety-related words | W = 361, uncorr. *p* = .129, FDR-corr. *p* = .268, *d* = 0.28 | W = 407, uncorr. *p* = .543, FDR-corr. *p* = .646, *d* = 0.08 | W = 392, uncorr. *p* = 1.000, FDR-corr. *p* = 1.000, *d* < 0.01 | W = 300, uncorr. *p* = .014, FDR-corr. *p* = .060, *d* = 0.54 | W = 1460, uncorr. *p* = .207, FDR-corr. *p* = .305, *d* = 0.19 |
| Sadness-related words | **W = 199, uncorr. *p* < .001, FDR-corr. *p* < .001, *d* = 1.24** | W = 364, uncorr. *p* = .322, FDR-corr. *p* = .423, *d* = 0.14 | W = 294, uncorr. *p* = .013, FDR-corr. *p* = .060, *d* = 0.51 | W = 362, uncorr. *p* = .162, FDR-corr. *p* = .270, *d* = 0.22 | **W = 1220, uncorr. *p* = .007, FDR-corr. *p* = .046, *d* = 0.50** |
| Subjective arousal [0-60 sec] | *t*(159) = -2.30, uncorr. *p* = .023, FDR-corr. *p* = .322, BF = 1.78, *d* = 0.60 | *t*(160) = -1.10, uncorr. *p* = .274, FDR-corr. *p* = .991, BF = 0.48, *d* = 0.29 | *t*(160) = -2.07, uncorr. *p* = .040, FDR-corr. *p* = .479, BF = 2.34, *d* = 0.55 | *t*(158) = -2.04, uncorr. *p* = .043, FDR-corr. *p* = .479, BF = 1.40, *d* = 0.54 | *t*(164) = -2.39, uncorr. *p* = .018, FDR-corr. *p* = .276, BF = 2.73, *d* = 0.50 |
| Subjective arousal [70-120 sec] | *t*(159) = -1.10, uncorr. *p* = .273, FDR-corr. *p* = .991, BF = 0.43, *d* = 0.29 | ***t*(158) = -0.04, uncorr. *p* = .968, FDR-corr. *p* = .991, BF = 0.27, *d* = 0.01** | *t*(161) = -1.06, uncorr. *p* = .291, FDR-corr. *p* = .991, BF = 0.46, *d* = 0.28 | *t*(157) = -1.49, uncorr. *p* = .139, FDR-corr. *p* = .991, BF = 0.77, *d* = 0.39 | *t*(164) = -1.18, uncorr. *p* = .240, FDR-corr. *p* = .991, BF = 0.40, *d* = 0.25 |
| Subjective arousal [130-180 sec] | *t*(159) = -1.43, uncorr. *p* = .156, FDR-corr. *p* = .991, BF = 0.62, *d* = 0.37 | *t*(158) = 0.71, uncorr. *p* = .481, FDR-corr. *p* = .991, BF = 0.33, *d* = 0.19 | ***t*(160) = -0.25, uncorr. *p* = .801, FDR-corr. *p* = .991, BF = 0.28, *d* = 0.07** | *t*(155) = -0.71, uncorr. *p* = .478, FDR-corr. *p* = .991, BF = 0.34, *d* = 0.19 | ***t*(163) = -0.55, uncorr. *p* = .583, FDR-corr. *p* = .991, BF = 0.25, *d* = 0.12** |
| BP [pre] | ***t*(163) = 0.30, uncorr. *p* = .761, FDR-corr. *p* = .991, BF = 0.29, *d* = 0.08** | ***t*(163) = -0.25, uncorr. *p* = .806, FDR-corr. *p* = .991, BF = 0.28, *d* = 0.07** | *t*(163) = 0.65, uncorr. *p* = .516, FDR-corr. *p* = .991, BF = 0.37, *d* = 0.17 | *t*(163) = 1.20, uncorr. *p* = .233, FDR-corr. *p* = .991, BF = 0.58, *d* = 0.32 | ***t*(166) = 0.60, uncorr. *p* = .546, FDR-corr. *p* = .991, BF = 0.28, *d* = 0.13** |
| BP [post] | ***t*(163) = 0.53, uncorr. *p* = .595, FDR-corr. *p* = .991, BF = 0.30, *d* = 0.14** | ***t*(163) = 0.41, uncorr. *p* = .685, FDR-corr. *p* = .991, BF = 0.29, *d* = 0.11** | ***t*(163) = 0.51, uncorr. *p* = .608, FDR-corr. *p* = .991, BF = 0.30, *d* = 0.14** | ***t*(163) = -0.11, uncorr. *p* = .909, FDR-corr. *p* = .991, BF = 0.27, *d* = 0.03** | ***t*(166) = 0.42, uncorr. *p* = .672, FDR-corr. *p* = .991, BF = 0.24, *d* = 0.09** |
| HR [0-60 sec] | *t*(56) = -0.23, uncorr. *p* = .818, FDR-corr. *p* = .991, BF = 0.42, *d* = 0.06 | *t*(86) = 0.14, uncorr. *p* = .887, FDR-corr. *p* = .991, BF = 0.37, *d* = 0.04 | *t*(79) = -0.44, uncorr. *p* = .661, FDR-corr. *p* = .991, BF = 0.52, *d* = 0.12 | *t*(111) = 0.34, uncorr. *p* = .733, FDR-corr. *p* = .991, BF = 0.36, *d* = 0.09 | ***t*(80) = -0.08, uncorr. *p* = .935, FDR-corr. *p* = .991, BF = 0.31, *d* = 0.02** |
| HR [70-120 sec] | *t*(64) = -0.28, uncorr. *p* = .784, FDR-corr. *p* = .991, BF = 0.39, *d* = 0.07 | *t*(88) = -0.02, uncorr. *p* = .986, FDR-corr. *p* = .991, BF = 0.34, *d* < 0.01 | *t*(88) = -0.89, uncorr. *p* = .374, FDR-corr. *p* = .991, BF = 0.93, *d* = 0.24 | ***t*(114) = -0.14, uncorr. *p* = .886, FDR-corr. *p* = .991, BF = 0.31, *d* = 0.04** | *t*(86) = -0.42, uncorr. *p* = .677, FDR-corr. *p* = .991, BF = 0.35, *d* = 0.09 |
| HR [130-180 sec] | *t*(67) = -0.08, uncorr. *p* = .937, FDR-corr. *p* = .991, BF = 0.35, *d* = 0.02 | *t*(90) = 0.03, uncorr. *p* = .979, FDR-corr. *p* = .991, BF = 0.34, *d* = 0.01 | *t*(91) = -0.65, uncorr. *p* = .520, FDR-corr. *p* = .991, BF = 0.58, *d* = 0.17 | ***t*(119) = 0.17, uncorr. *p* = .862, FDR-corr. *p* = .991, BF = 0.31, *d* = 0.05** | ***t*(89) = -0.17, uncorr. *p* = .867, FDR-corr. *p* = .991, BF = 0.29, *d* = 0.04** |
| SC [0-60 sec] | *t*(90) = 1.34, uncorr. *p* = .183, FDR-corr. *p* = .991, BF = 1.68, *d* = 0.35 | *t*(69) = 1.35, uncorr. *p* = .180, FDR-corr. *p* = .991, BF = 8.29, *d* = 0.36 | *t*(80) = 0.61, uncorr. *p* = .543, FDR-corr. *p* = .991, BF = 0.71, *d* = 0.16 | *t*(106) = -0.30, uncorr. *p* = .762, FDR-corr. *p* = .991, BF = 0.36, *d* = 0.08 | *t*(88) = 0.98, uncorr. *p* = .332, FDR-corr. *p* = .991, BF = 1.19, *d* = 0.21 |
| SC [70-120 sec] | *t*(77) = 0.40, uncorr. *p* = .688, FDR-corr. *p* = .991, BF = 0.38, *d* = 0.11 | *t*(76) = 0.82, uncorr. *p* = .416, FDR-corr. *p* = .991, BF = 1.18, *d* = 0.22 | *t*(79) = 0.07, uncorr. *p* = .943, FDR-corr. *p* = .991, BF = 0.37, *d* = 0.02 | *t*(91) = -0.97, uncorr. *p* = .335, FDR-corr. *p* = .991, BF = 1.28, *d* = 0.26 | *t*(81) = 0.12, uncorr. *p* = .908, FDR-corr. *p* = .991, BF = 0.32, *d* = 0.02 |
| SC [130-180 sec] | *t*(90) = 0.24, uncorr. *p* = .813, FDR-corr. *p* = .991, BF = 0.37, *d* = 0.06 | *t*(67) = 0.66, uncorr. *p* = .513, FDR-corr. *p* = .991, BF = 0.91, *d* = 0.18 | *t*(73) = 0.14, uncorr. *p* = .888, FDR-corr. *p* = .991, BF = 0.46, *d* = 0.04 | *t*(86) = -0.43, uncorr. *p* = .671, FDR-corr. *p* = .991, BF = 0.54, *d* = 0.11 | *t*(86) = 0.21, uncorr. *p* = .836, FDR-corr. *p* = .991, BF = 0.33, *d* = 0.04 |
| PEP [0-60 sec] | *t*(39) = -0.19, uncorr. *p* = .852, FDR-corr. *p* = .991, BF = 0.52, *d* = 0.05 | *t*(79) = 0.01, uncorr. *p* = .990, FDR-corr. *p* = .991, BF = 0.40, *d* < 0.01 | *t*(72) = -0.48, uncorr. *p* = .634, FDR-corr. *p* = .991, BF = 0.66, *d* = 0.13 | *t*(107) = -0.35, uncorr. *p* = .727, FDR-corr. *p* = .991, BF = 0.40, *d* = 0.09 | *t*(69) = -0.31, uncorr. *p* = .755, FDR-corr. *p* = .991, BF = 0.36, *d* = 0.07 |
| PEP [70-120 sec] | *t*(42) = -0.15, uncorr. *p* = .881, FDR-corr. *p* = .991, BF = 0.55, *d* = 0.04 | *t*(80) = -0.07, uncorr. *p* = .943, FDR-corr. *p* = .991, BF = 0.38, *d* = 0.02 | *t*(73) = -0.40, uncorr. *p* = .688, FDR-corr. *p* = .991, BF = 0.63, *d* = 0.11 | *t*(105) = -0.16, uncorr. *p* = .876, FDR-corr. *p* = .991, BF = 0.36, *d* = 0.04 | *t*(69) = -0.25, uncorr. *p* = .807, FDR-corr. *p* = .991, BF = 0.36, *d* = 0.05 |
| PEP [130-180 sec] | *t*(43) = -0.51, uncorr. *p* = .610, FDR-corr. *p* = .991, BF = 1.01, *d* = 0.13 | *t*(85) = 0.13, uncorr. *p* = .900, FDR-corr. *p* = .991, BF = 0.42, *d* = 0.03 | *t*(71) = -0.37, uncorr. *p* = .715, FDR-corr. *p* = .991, BF = 0.55, *d* = 0.10 | *t*(101) = -0.11, uncorr. *p* = .912, FDR-corr. *p* = .991, BF = 0.35, *d* = 0.03 | *t*(71) = -0.31, uncorr. *p* = .755, FDR-corr. *p* = .991, BF = 0.37, *d* = 0.07 |
| RR [0-60 sec] | ***t*(126) = -0.08, uncorr. *p* = .939, FDR-corr. *p* = .991, BF = 0.29, *d* = 0.02** | *t*(148) = 1.01, uncorr. *p* = .315, FDR-corr. *p* = .991, BF = 0.42, *d* = 0.27 | *t*(156) = 1.07, uncorr. *p* = .286, FDR-corr. *p* = .991, BF = 0.44, *d* = 0.29 | *t*(153) = 1.61, uncorr. *p* = .109, FDR-corr. *p* = .991, BF = 0.77, *d* = 0.43 | *t*(148) = 1.10, uncorr. *p* = .271, FDR-corr. *p* = .991, BF = 0.41, *d* = 0.23 |
| RR [70-120 sec] | *t*(123) = -2.11, uncorr. *p* = .037, FDR-corr. *p* = .473, BF = 15.99, *d* = 0.56 | *t*(146) = -0.93, uncorr. *p* = .352, FDR-corr. *p* = .991, BF = 0.43, *d* = 0.25 | *t*(155) = -0.80, uncorr. *p* = .426, FDR-corr. *p* = .991, BF = 0.42, *d* = 0.21 | ***t*(151) = -0.46, uncorr. *p* = .646, FDR-corr. *p* = .991, BF = 0.31, *d* = 0.12** | *t*(147) = -1.39, uncorr. *p* = .166, FDR-corr. *p* = .991, BF = 0.62, *d* = 0.29 |
| RR [130-180 sec] | ***t*(127) = 0.01, uncorr. *p* = .991, FDR-corr. *p* = .991, BF = 0.30, *d* < 0.01** | *t*(146) = 0.91, uncorr. *p* = .365, FDR-corr. *p* = .991, BF = 0.44, *d* = 0.24 | *t*(155) = 0.82, uncorr. *p* = .414, FDR-corr. *p* = .991, BF = 0.38, *d* = 0.22 | *t*(151) = 1.53, uncorr. *p* = .128, FDR-corr. *p* = .991, BF = 0.98, *d* = 0.41 | *t*(147) = 1.01, uncorr. *p* = .316, FDR-corr. *p* = .991, BF = 0.38, *d* = 0.21 |
| RSA [0-60 sec] | *t*(106) = 0.33, uncorr. *p* = .739, FDR-corr. *p* = .991, BF = 0.32, *d* = 0.09 | ***t*(135) = -0.35, uncorr. *p* = .729, FDR-corr. *p* = .991, BF = 0.30, *d* = 0.09** | *t*(151) = 0.87, uncorr. *p* = .388, FDR-corr. *p* = .991, BF = 0.40, *d* = 0.23 | *t*(147) = -0.94, uncorr. *p* = .349, FDR-corr. *p* = .991, BF = 0.42, *d* = 0.25 | ***t*(138) = -0.02, uncorr. *p* = .983, FDR-corr. *p* = .991, BF = 0.24, *d* < 0.01** |
| RSA [70-120 sec] | *t*(93) = 0.32, uncorr. *p* = .748, FDR-corr. *p* = .991, BF = 0.32, *d* = 0.08 | ***t*(122) = 0.09, uncorr. *p* = .928, FDR-corr. *p* = .991, BF = 0.30, *d* = 0.02** | *t*(143) = 1.47, uncorr. *p* = .145, FDR-corr. *p* = .991, BF = 0.40, *d* = 0.39 | *t*(136) = -0.25, uncorr. *p* = .803, FDR-corr. *p* = .991, BF = 0.42, *d* = 0.07 | ***t*(128) = 0.50, uncorr. *p* = .619, FDR-corr. *p* = .991, BF = 0.24, *d* = 0.11** |
| RSA [130-180 sec] | *t*(111) = 1.05, uncorr. *p* = .297, FDR-corr. *p* = .991, BF = 0.61, *d* = 0.28 | *t*(136) = 0.73, uncorr. *p* = .464, FDR-corr. *p* = .991, BF = 0.37, *d* = 0.20 | *t*(152) = 1.53, uncorr. *p* = .127, FDR-corr. *p* = .991, BF = 0.80, *d* = 0.41 | ***t*(146) = 0.26, uncorr. *p* = .797, FDR-corr. *p* = .991, BF = 0.29, *d* = 0.07** | *t*(138) = 1.13, uncorr. *p* = .262, FDR-corr. *p* = .991, BF = 0.54, *d* = 0.24 |
| RSA (corrected for RR) [0-60 sec] | *t*(97) = 0.34, uncorr. *p* = .737, FDR-corr. *p* = .991, BF = 0.61, *d* = 0.09 | *t*(128) = 0.44, uncorr. *p* = .662, FDR-corr. *p* = .991, BF = 0.37, *d* = 0.12 | *t*(147) = 1.33, uncorr. *p* = .186, FDR-corr. *p* = .991, BF = 0.80, *d* = 0.36 | ***t*(142) = 0.11, uncorr. *p* = .914, FDR-corr. *p* = .991, BF = 0.29, *d* = 0.03** | *t*(129) = 0.68, uncorr. *p* = .498, FDR-corr. *p* = .991, BF = 0.54, *d* = 0.14 |
| RSA (corrected for RR) [70-120 sec] | *t*(106) = 0.20, uncorr. *p* = .841, FDR-corr. *p* = .991, BF = 0.32, *d* = 0.05 | *t*(140) = -1.20, uncorr. *p* = .230, FDR-corr. *p* = .991, BF = 0.62, *d* = 0.32 | ***t*(152) = 0.31, uncorr. *p* = .760, FDR-corr. *p* = .991, BF = 0.29, *d* = 0.08** | *t*(146) = -0.74, uncorr. *p* = .462, FDR-corr. *p* = .991, BF = 0.38, *d* = 0.20 | ***t*(140) = -0.44, uncorr. *p* = .664, FDR-corr. *p* = .991, BF = 0.26, *d* = 0.09** |
| RSA (corrected for RR) [130-180 sec] | *t*(98) = 0.22, uncorr. *p* = .830, FDR-corr. *p* = .991, BF = 0.32, *d* = 0.06 | *t*(128) = -0.92, uncorr. *p* = .358, FDR-corr. *p* = .991, BF = 0.62, *d* = 0.25 | ***t*(147) = 0.63, uncorr. *p* = .528, FDR-corr. *p* = .991, BF = 0.29, *d* = 0.17** | *t*(138) = -0.20, uncorr. *p* = .840, FDR-corr. *p* = .991, BF = 0.38, *d* = 0.05 | ***t*(132) = -0.08, uncorr. *p* = .936, FDR-corr. *p* = .991, BF = 0.26, *d* = 0.02** |
| CO [0-60 sec] | *t*(55) = -0.06, uncorr. *p* = .953, FDR-corr. *p* = .991, BF = 0.44, *d* = 0.02 | *t*(82) = 0.03, uncorr. *p* = .973, FDR-corr. *p* = .991, BF = 0.40, *d* = 0.01 | *t*(76) = -0.35, uncorr. *p* = .729, FDR-corr. *p* = .991, BF = 0.54, *d* = 0.09 | *t*(108) = 0.17, uncorr. *p* = .862, FDR-corr. *p* = .991, BF = 0.36, *d* = 0.05 | ***t*(84) = -0.07, uncorr. *p* = .943, FDR-corr. *p* = .991, BF = 0.30, *d* = 0.02** |
| CO [70-120 sec] | *t*(64) = 0.04, uncorr. *p* = .964, FDR-corr. *p* = .991, BF = 0.37, *d* = 0.01 | *t*(94) = 0.04, uncorr. *p* = .968, FDR-corr. *p* = .991, BF = 0.36, *d* = 0.01 | *t*(85) = -0.72, uncorr. *p* = .471, FDR-corr. *p* = .991, BF = 0.82, *d* = 0.19 | *t*(113) = -0.02, uncorr. *p* = .984, FDR-corr. *p* = .991, BF = 0.32, *d* = 0.01 | ***t*(93) = -0.21, uncorr. *p* = .837, FDR-corr. *p* = .991, BF = 0.29, *d* = 0.04** |
| CO [130-180 sec] | *t*(58) = 0.30, uncorr. *p* = .764, FDR-corr. *p* = .991, BF = 0.42, *d* = 0.08 | *t*(89) = 0.22, uncorr. *p* = .824, FDR-corr. *p* = .991, BF = 0.41, *d* = 0.06 | *t*(82) = -0.49, uncorr. *p* = .626, FDR-corr. *p* = .991, BF = 0.52, *d* = 0.13 | *t*(110) = 0.21, uncorr. *p* = .833, FDR-corr. *p* = .991, BF = 0.34, *d* = 0.06 | ***t*(90) = 0.09, uncorr. *p* = .928, FDR-corr. *p* = .991, BF = 0.28, *d* = 0.02** |
| TPR [0-60 sec] | *t*(73) = 0.35, uncorr. *p* = .727, FDR-corr. *p* = .991, BF = 0.40, *d* = 0.09 | ***t*(121) = -0.14, uncorr. *p* = .889, FDR-corr. *p* = .991, BF = 0.31, *d* = 0.04** | *t*(103) = 0.82, uncorr. *p* = .414, FDR-corr. *p* = .991, BF = 0.74, *d* = 0.22 | *t*(128) = 0.65, uncorr. *p* = .519, FDR-corr. *p* = .991, BF = 0.50, *d* = 0.17 | *t*(114) = 0.55, uncorr. *p* = .585, FDR-corr. *p* = .991, BF = 0.34, *d* = 0.12 |
| TPR [70-120 sec] | *t*(67) = 0.58, uncorr. *p* = .563, FDR-corr. *p* = .991, BF = 0.45, *d* = 0.15 | *t*(114) = 0.05, uncorr. *p* = .958, FDR-corr. *p* = .991, BF = 0.31, *d* = 0.01 | *t*(102) = 0.99, uncorr. *p* = .322, FDR-corr. *p* = .991, BF = 1.19, *d* = 0.27 | *t*(128) = 0.53, uncorr. *p* = .599, FDR-corr. *p* = .991, BF = 0.39, *d* = 0.14 | *t*(103) = 0.70, uncorr. *p* = .487, FDR-corr. *p* = .991, BF = 0.46, *d* = 0.15 |
| TPR [130-180 sec] | *t*(70) = 0.31, uncorr. *p* = .756, FDR-corr. *p* = .991, BF = 0.37, *d* = 0.08 | *t*(112) = -0.17, uncorr. *p* = .867, FDR-corr. *p* = .991, BF = 0.32, *d* = 0.04 | *t*(98) = 0.55, uncorr. *p* = .580, FDR-corr. *p* = .991, BF = 0.46, *d* = 0.15 | *t*(123) = -0.30, uncorr. *p* = .766, FDR-corr. *p* = .991, BF = 0.33, *d* = 0.08 | ***t*(102) = 0.15, uncorr. *p* = .882, FDR-corr. *p* = .991, BF = 0.27, *d* = 0.03** |

*Note*. Based on linear models. Results with significant *p*-values after FDR-correction are displayed in bold black font, and results with substantial Bayesian evidence for the null hypothesis are displayed in bold orange font, with inconclusive results in regular font. FDR = false-discovery rate; BF = Bayes Factor; *d* = Cohen’s *d*; BP = blood pressure; HR = heart rate; SC = skin conductance; PEP = pre-ejection period; RR = respiratory rate; RSA = respiratory sinus arrhythmia; CO = cardiac output; TPR = total peripheral resistance.

Table S4. Correlations between reactivity of affect, subjective arousal, physiological activation, and personality traits.

*Note*. Results with significant *p*-values after FDR-correction are displayed in bold black font, and results with substantial Bayesian evidence for the null hypothesis are displayed in bold orange font, with inconclusive results in regular font. *r* = Pearson correlation coefficient; FDR = false-discovery rate; BF = Bayes Factor; *d* = Cohen’s *d*; subj. = subjective; BP = blood pressure; HR = heart rate; SC = skin conductance; PEP = pre-ejection period; RR = respiratory rate; RSA = respiratory sinus arrhythmia; CO = cardiac output; TPR = total peripheral resistance.

**References**

Debski, T. T., Zhang, Y., Jennings, J. R., and Kamarck, T. W. (1993). Stability of cardiac impedance measures: Aortic opening (B-point) detection and scoring. *Biol. Psychol.* 36, 63–74. doi:10.1016/0301-0511(93)90081-I.

Seery, M. D., Kondrak, C. L., Streamer, L., Saltsman, T., and Lamarche, V. M. (2016). Preejection period can be calculated using R peak instead of Q. *Psychophysiology* 53, 1232–1240. doi:10.1111/psyp.12657.

Sherwood, A., Allen, M. T., Fahrenberg, J., Kelsey, R. M., Lovallo, W. R., and Doornen, L. J. P. (1990). Methodological guidelines for impedance cardiography. *Psychophysiology* 27, 1–23. doi:10.1111/j.1469-8986.1990.tb02171.x.

Sjak-Shie, E. E. (2017). PhysioData Toolbox.
